# Imaging Mass Spectrometry Reveals Tumor Metabolic Heterogeneity

**DOI:** 10.1016/j.isci.2020.101355

**Published:** 2020-07-10

**Authors:** Yang Zhang, Christelle Guillermier, Thomas De Raedt, Andrew G. Cox, Ophelia Maertens, Dean Yimlamai, Mingyue Lun, Adam Whitney, Richard L. Maas, Wolfram Goessling, Karen Cichowski, Matthew L. Steinhauser

**Affiliations:** 1 Department of Medicine, Division of Genetics, Brigham and Women's Hospital, Boston, MA, USA; 2 Harvard Medical School, Boston, MA, USA; 3 Aging Institute, University of Pittsburgh School of Medicine, Pittsburgh, PA, USA; 4 Boston Children's Hospital, Boston, MA, USA; 5 Ludwig Center, Dana-Farber/Harvard Cancer Center, Boston, MA, USA

**Keywords:** Biological Sciences, Cancer Systems Biology

## Abstract

Malignant tumors exhibit high degrees of genomic heterogeneity at the cellular level, leading to the view that subpopulations of tumor cells drive growth and treatment resistance. To examine the degree to which tumors also exhibit metabolic heterogeneity at the level of individual cells, we employed multi-isotope imaging mass spectrometry (MIMS) to quantify utilization of stable isotopes of glucose and glutamine along with a label for cell division. Mouse models of melanoma and malignant peripheral nerve sheath tumors (MPNSTs) exhibited striking heterogeneity of substrate utilization, evident in both proliferating and non-proliferating cells. We identified a correlation between metabolic heterogeneity, proliferation, and therapeutic resistance. Heterogeneity in metabolic substrate usage as revealed by incorporation of glucose and glutamine tracers is thus a marker for tumor proliferation. Collectively, our data demonstrate that MIMS provides a powerful tool with which to dissect metabolic functions of individual cells within the native tumor environment.

## Highlights

• A method to measure substrate utilization at single cancer cell resolution
• Heterogeneity of glucose and glutamine utilization in murine tumors
• Metabolic heterogeneity in proliferating and non-proliferating tumor cells
• Metabolic heterogeneity correlates with proliferative growth and treatment resistance

## Graphical Abstract

**Figure:**
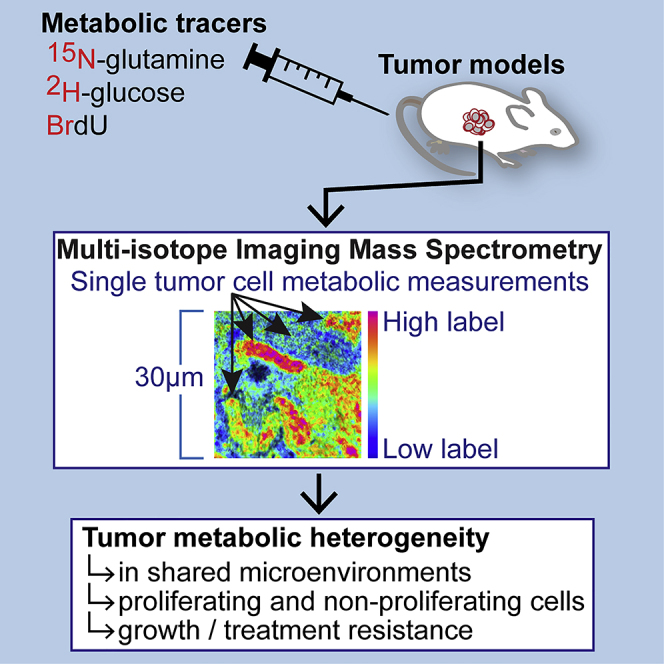

## Introduction

Cancer cells undergo metabolic changes that promote anabolic growth and proliferation (Vander Heiden and DeBerardinis, 2017). Positron emission tomographic (PET) imaging of metabolic radiotracers, such as ^18^F-2-deoxyglucose (FDG), leverages the heightened metabolic activity of cancer cells to define local and metastatic disease (Fletcher et al., 2008; Juweid and Cheson, 2006; Kelloff et al., 2005; Torizuka et al., 1995; Weber et al., 1999). In addition, the relative intensity and/or heterogeneity of FDG-uptake within a tumor may inform prognosis beyond characterization of disease burden (Eary et al., 2008; Juweid and Cheson, 2006; Kang et al., 2014; Kidd and Grigsby, 2008; Yoon et al., 2015). Such regions of intratumor variability in FDG-PET avidity are also associated with differential metabolic flux of stable isotope metabolic tracers and therefore indicate true functional heterogeneity (Faubert et al., 2017; Hensley et al., 2016). These data suggest that the paradigm of tumor heterogeneity—well recognized at the molecular level (Anderson et al., 2011; Azizi et al., 2018; Jackson et al., 2020; Patel et al., 2014; Tirosh et al., 2016)—also extends to the metabolic function of tumor cells.

Various aspects of tumor metabolic function have been interrogated by magnetic resonance (MR) spectroscopy, mass spectrometry, or PET imaging, using a range of radiolabeled or stable isotope-labeled substrates of metabolic pathways related to cellular proliferation, glucose metabolism, or amino acid metabolism (Fan et al., 2009; Hensley et al., 2016; Maher et al., 2012; Sengupta and Pratx, 2016). However, existing approaches to assess metabolic functions of tumors generally do so at tissue-scale resolution. As such, the cellular basis for signals of metabolic heterogeneity in tumors has not been completely elucidated. With FDG-PET imaging of tumor metabolic heterogeneity, for example, differential FDG uptake could in theory arise from cell autonomous differences in tumor metabolic programming or simply from regional differences in cell viability, tumor cell proliferation, or other effects related to averaging signal within arbitrary voxels encompassing millimeter to centimeter swaths of tissue (Brooks, 2013).

Defining tumor metabolic functions at the resolution of individual cells, particularly within the native tumor microenvironment, has lagged behind what is achievable with multidimensional biomarker analyses (e.g., mass cytometry) and with single cell genomics (Anderson et al., 2011; Keren et al., 2018; Patel et al., 2014). Historically, the highest resolution approach to studying metabolic functions of tissues involved autoradiographic detection of radiolabeled tracers coupled with electron microscopy (Coimbra and Leblond, 1966). Newer metabolic imaging approaches, such as the imaging of novel fluorescent metabolic tracers, imaging of cellular redox states by their autofluorescence properties, or imaging of tracers with Raman scattering microscopy, do not achieve the lateral resolution of electron microscopy but provide images of metabolic activities with sufficient spatial resolution to capture individual cells (Hu et al., 2015; O'Neil et al., 2005; Walsh et al., 2014; Wei et al., 2013). These methods, which are at various stages of development, are generally semi-quantitative.

A new form of imaging mass spectrometry, multi-isotope imaging mass spectrometry or MIMS, can be used to quantify stable isotope incorporation at suborganelle resolution (Lechene et al., 2006; Steinhauser et al., 2012). Central to MIMS is a nanoscale secondary ion mass spectrometry (NanoSIMS) instrument, which probes a sample surface with an ion beam at a lateral resolution of down to 30 nm, resulting in ionization of the uppermost atoms and polyatomic fragments (Gyngard and Steinhauser, 2019). The yield of negatively charged secondary ions are extracted and separated in a magnetic sector. Seven detectors are aligned to quantify up to seven discrete ionic species in parallel. When two detectors are aligned to capture isotopic variants of the same element, the incorporation of tracers tagged with rare stable isotopic variants (e.g., ^2^H, ^13^C, ^15^N) can be quantified by the corresponding increase in the isotopic ratio. Advantages of using stable isotope tracers for metabolic analyses include: (1) they seamlessly integrate into biochemical pathways because introduction of an isotopic variant does not alter the molecular structure of the parent compound, (2) they do not confer toxicity in the manner of fluorescent tags or radiolabels, and (3) they are quantifiable with high accuracy by mass spectrometry methods, including NanoSIMS. Therefore, MIMS merges imaging at high spatial resolution with the quantitative power of isotope ratio mass spectrometry.

Prior applications of MIMS to non-cancerous tissues has provided a framework to quantify stable isotope-tagged glucose, amino acids, and precursors of nucleic acid synthesis in a multiplexed fashion (Guillermier et al., 2017b, 2019; Steinhauser et al., 2012; Zhang et al., 2012). We reasoned that MIMS could also enable quantitative measurement of substrate utilization in individual cancer cells, thereby enabling us to test the hypothesis that tumors exhibit metabolic heterogeneity at the level of individual cancer cells. Here, we present the first application of MIMS to interrogate metabolic heterogeneity of tumors *in vivo.* In mouse models of melanoma and malignant peripheral nerve sheath tumors (MPNSTs), we discovered striking heterogeneity of substrate utilization. Moreover, in an MPNST model, we identified a strong correlation between metabolic heterogeneity, proliferation, and therapeutic resistance.

## Results

### Heterogeneity of Glucose and Glutamine Utilization by Proliferating Cancer Cells

**Figure 1:**
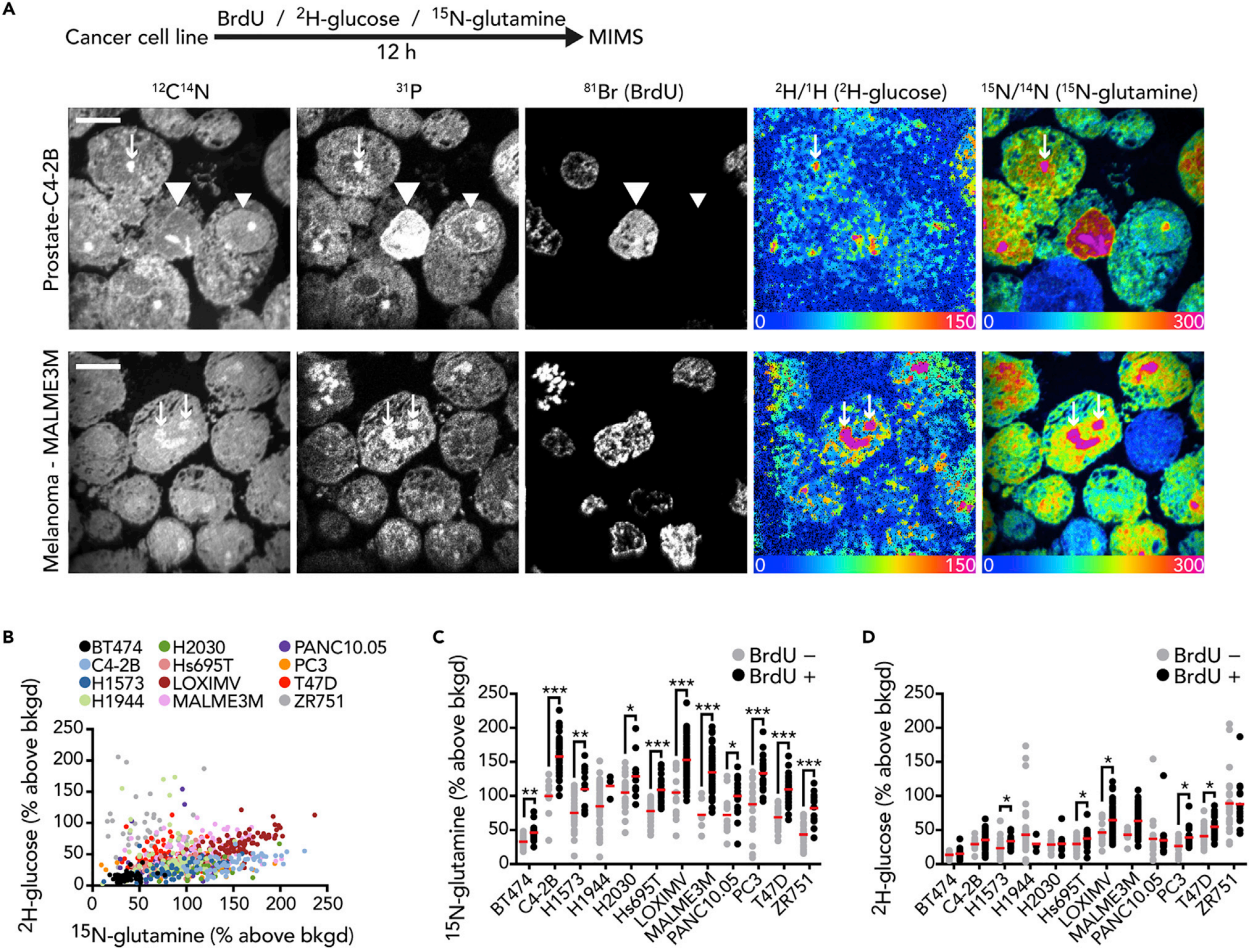
Heterogeneity of Glucose and Glutamine Utilization by Proliferating Cancer Cells (A) Cancer cell lines were labeled with a cocktail consisting of ^2^H-glucose, ^15^N-glutamine, and bromodeoxyuridine (BrdU) for 12 h. Two representative cell lines are shown: MALME3M (melanoma) and C4-2B (prostate). ^12^C^14^N and ^31^P mass images reveal cellular borders and details such as nuclei. BrdU incorporation by cells that divided during the labeling period is indicated by direct measurement of ^81^Br into nuclei that are also evident in the ^12^C^14^N and ^31^P mass images (example: large arrow heads). An adjacent BrdU^−^ cell is indicated by a small arrow head. Hue saturation intensity (HSI) images display the isotope ratio measurements and therefore a map of the incorporation of ^2^H-glucose and ^15^N-glutamine. Arrows indicate metabolic labeling hotspots with features consistent with nucleoli in the ^12^C^14^N and ^31^P mass images. The lower bound of the scale (blue) is set to the background ratio (0%) and the upper bound (magenta) is set to reveal differences in labeling (150% and 300% above background, respectively). Scale bar, 10 μm. (B) Dot plot of individual cancer cells, color coded according to cell line (legend). ^15^N-glutamine and ^2^H-glucose labeling expressed as percentage above natural background. (C) ^15^N-glutamine labeling of cancer cell lines as a function of BrdU labeling (divided cells are BrdU^+^). (D) ^2^H-glucose labeling of cancer cell lines as a function of BrdU labeling. For (C) and (D): red line = mean; ∗p < 0.05, ∗∗p < 0.001, ∗∗∗p < 0.0001, two-tailed t test. See also Figure S2 and S3.

The application of FDG-glucose—and more recently labeled glutamine (Salamanca-Cardona et al., 2017; Venneti et al., 2015)—to tumor imaging is driven by the observation that proliferating cancer cells coopt glucose and glutamine as substrates for anabolic growth. These observations provided a rationale for using stable isotope-tagged glucose and glutamine as metabolic labels for MIMS, which we used together with Bromodeoxyuridine (BrdU) as a nucleotide label for cell division (Figure S1, see also Transparent Methods in Supplemental Information). We selected ^2^H- rather than ^13^C-glucose, because the signal to background characteristics of ^13^C are less desirable owing to its relatively high background concentration in embedded samples relative to ^2^H (Gyngard and Steinhauser, 2019). We first tested this approach in cancer cell lines labeled for 12 h prior to MIMS analysis (Figure 1A). Images of CN^−^ and P^−^ intensity delineated cell and nuclear borders as we have previously shown (Kim et al., 2014; Steinhauser et al., 2012) and guided the extraction of quantitative labeling data. We measured ^2^H-glucose and ^15^N-glutamine labels by an increase in the respective isotope ratios above natural background: specifically, ^2^H-labeling by an increase in the ^12^C_2_^2^H^−^/^12^C_2_^1^H^−^ ratio and ^15^N-labeling by an increase in the ^12^C^15^N^−^/^12^C^14^N^−^ ratio (Figures 1A and S1) (Guillermier et al., 2017b; Steinhauser et al., 2012). Such increases in labeling are visually represented by a hue saturation intensity (HSI) transformation, where the blue end of the scale is set at natural abundance and the upper magenta bound of the scale is set to reveal labeling differences. Importantly, scaling changes modify the visual representation; however, the underlying quantitative data that are extracted for each region of interest (ROI) remain unmodified. An additional feature of HSI images is that the pixel intensity reflects the number of ion counts and as such a pixel with low counts will appear dark. This is particularly relevant to the ^2^H measurements, because the electron affinity and hence yield of C_2_H^−^ ions is low relative to CN^−^, the ionic species used for ^15^N measurements. This difference in electron affinity accounts for some of the ^2^H-glucose images appearing dark, particularly at the margins of the imaging field. Although low ion counts limit statistical conclusions from an individual pixel, in the current application where the selected ROIs are relatively large structures (e.g., whole cells), any given data point is calculated by merging the ion counts from the numerous pixels contained within the ROI. As such, regions that appear dark in the HSI image may still provide isotope ratio data (Figure S1B). In contrast to stable isotope tracers, incorporation of BrdU in the nucleus of dividing cells is detectable by direct measurement of Br^−^ intensity (Steinhauser et al., 2012). We observed variability in ^2^H-glucose and ^15^N-glutamine labeling between and within cell lines, spanning 1–2 orders of magnitude in intensity (Figure 1B). For most of the cell lines, we observed a significant increase in the distribution of glucose and/or glutamine labeling in the BrdU^+^ fraction relative to cells that remained BrdU^−^, consistent with utilization of glucose and glutamine by cancer cells as substrate for growth.

MIMS measures isotope ratios at the elemental level without molecular specificity, and therefore, an increased signal may represent incorporation of the parent tracer molecule by different pathways, which is of relevance to the current application given that both ^2^H-glucose and ^15^N-glutamine are metabolized to diverse fates intracellularly (De Feyter et al., 2018; Xu et al., 2016). For ^2^H-glucose, it is also possible that some signal is diluted by proton exchange. Moreover, MIMS sample preparation involves standard histological procedures including fixation and dehydration prior to embedding, and therefore, freely diffusible parent tracer molecules and/or downstream metabolites are lost prior to MIMS analysis. In order to explore these modifying factors, we performed *in vitro* stable isotope labeling studies and performed bulk isotope ratio mass spectrometry (IRMS) to assess how sample processing affects signal and how the signal may reflect a variety of metabolic fates. We selected IRMS because it is higher throughput than MIMS, particularly when testing a range of experimental conditions, while providing similarly precise isotope ratio measurements. For these experiments, we utilized ^13^C as a glucose label rather than ^2^H because our IRMS analysis enables concomitant measurement of carbon and nitrogen isotope ratios from the same sample. We first tested the degree to which fixation, which disrupts membranes, and subsequent washing diluted labeling. In the two cell lines examined (LOXIMV1 and MALME3M), processing cells for MIMS resulted in loss of signal relative to unprocessed cells; however, approximately 75% of the glucose signal and approximately 90% of the glutamine signal was retained during sample processing (Figures S3A and S3B). Protein synthesis and nucleic acid synthesis are two key components of anabolic growth, and their biosynthetic products subject to aldehyde fixation, and hence part of the fixable biomass of the cell. Indeed, when we isolated the protein, DNA, and RNA from cells labeled with ^13^C-glucose and ^15^N-glutamine, we detected label incorporation into all three fractions (Figures S3C and S3D). Relative to unprocessed whole cells, the nucleic acid signal was equal or higher than unprocessed whole cells for both glucose and glutamine, consistent with contribution of these substrates to nucleic acid synthesis. Incorporation of both labels into the protein fraction was also detectable, although the contribution of ^13^C-glucose to protein was smaller relative to the labeled amino acid ^15^N-glutamine. The contribution of ^13^C-glucose to biosynthetic reactions and the cellular biomass was further demonstrated by comparing labeling by ^13^C-glucose to ^13^C-2-deoxy-d-glucose, which like FDG-glucose, is taken up by cells but cannot participate in glycolysis (Figure S3E). Not surprisingly, ^13^C-glucose labeling was consistently higher than that achieved with ^13^C-2-deoxy-d-glucose, consistent with both its cellular uptake and incorporation into cellular biomass. Collectively, these data support the concept that MIMS measurement of glucose and glutamine labeling indicates incorporation of the parent molecule or its metabolites into the fixable cellular biomass, including pathways critical to growth such as protein and nucleic acid synthesis. Incorporation of label may reflect direct utilization of the parent molecule by anabolic reactions, for example, the incorporation of labeled amino acids into newly synthesized protein. Alternatively, the parent molecules may be first utilized by various reactions, including catabolic reactions, with downstream labeled metabolites providing substrate for an anabolic reaction. Therefore, for the purposes of this manuscript, we apply the term *utilization* as a descriptor of tracer incorporation arising from anabolic reactions, catabolic reactions, or some combination of the two.

In these analyses, each data point represented an isotopic measurement of the cellular material contained within the circumference of the cell, which is a merger of the data of the pixels contained within the cell border. The measurement still represents a sampling of the cell, however, because only the uppermost atomic layers are sputtered and ionized (<1 nm). Therefore, we examined the degree to which an assessment of heterogeneity would be modified by increasing our resolution to focus on a discrete population of organelles. We used the BrdU labeling images to precisely define a population of BrdU^+^ nuclei and then compared the glucose and glutamine labeling distributions with those obtained from the corresponding whole-cell analyses inclusive of the nuclei and other intracellular structures (Figure S2). In order to assess relative degrees of heterogeneity between the two distributions, we employed the median absolute deviation (MAD), which we selected as a robust indicator of dispersion that incorporates all data points, which can be applied to normal and non-normal distributions and which remains resilient to outliers because all data points are weighted equally (Hampel, 1974; Martin and Zamar, 1989). For most of the cell lines, the metric of heterogeneity increased as we focused the analysis on the nucleus rather than the entire cell (Figure S2). These data are consistent with the concept that, as resolution increases, there tends to be a corresponding increase in observed heterogeneity.

### Heterogeneity of Glucose and Glutamine Utilization in Murine Tumors

**Figure 2:**
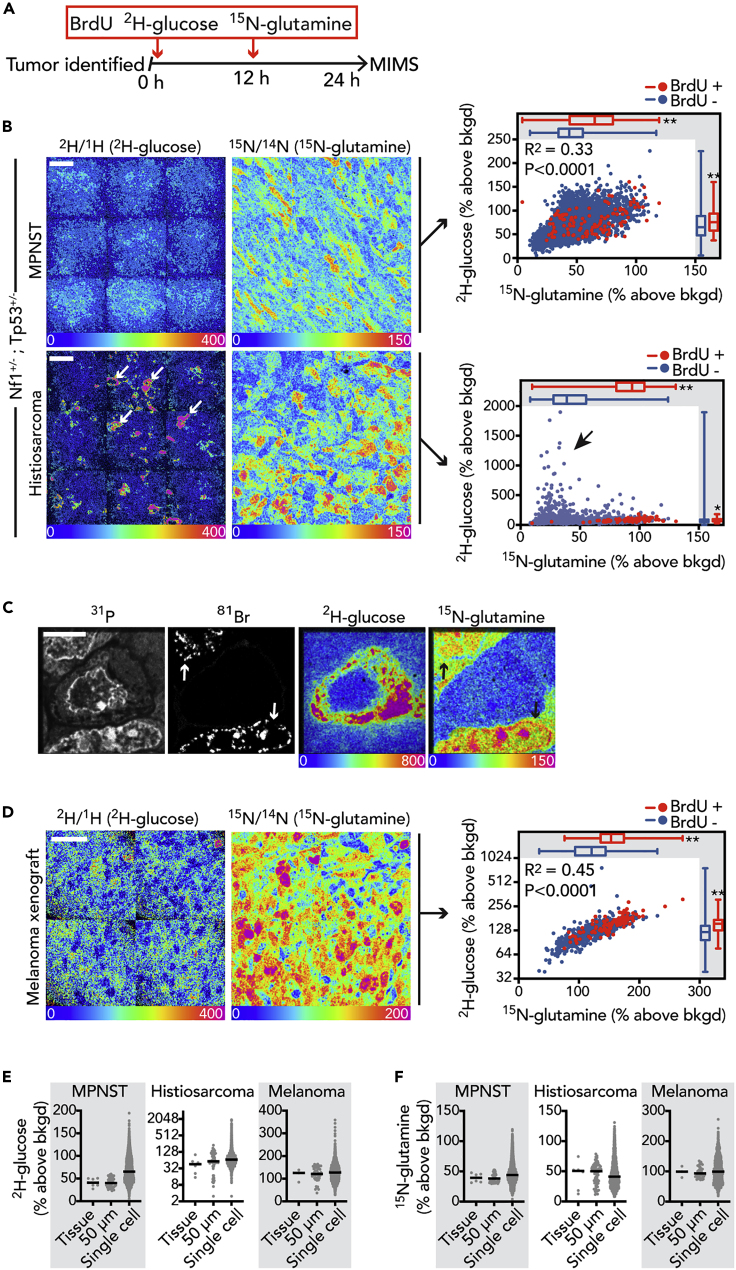
Heterogeneity of Glucose and Glutamine Utilization in Murine Tumors (A) Schematic depicting labeling protocol. (B) Tumor bearing *Nf1*^*+/−*^*;Tp53*^*+/−*^ mouse with palpable subcutaneous MPNST was labeled (top). A second tumor (histiosarcoma, bottom) was identified incidentally in the abdomen. In these representative HSI images, adjacent imaging fields (50 μm × 50 μm) are tiled together as a mosaic image to cover a larger swath of tissue. The heterogeneity of glucose and glutamine avidity is shown quantitatively in the respective dot plots on the right, where BrdU^+^ cells are red. Arrows identify cells that are intensely ^2^H-glucose labeled in the histiosarcoma and that correspond to the glucose-high/glutamine-low cells in the graph on the right (box). Univariate comparisons between BrdU^−^ (blue) and BrdU^+^ (red) cells are shown by the box and whisker plots embedded in the graphs: ∗p < 0.0005, ∗∗p < 0.0001, Mann-Whitney test. Scale bar, 20 μm. (C) Higher-resolution imaging of a single representative glucose-high, glutamine-low cell surrounded by two glucose-low, glutamine-high cells, which are also BrdU^+^ (arrows). Note: ^15^N-glutamine hotspots in the nucleus co-localizing with regions of BrdU incorporation. Scale bar, 5 μm. (D) HSI images of melanoma xenograft. Right: heterogeneity of ^2^H-glucose and ^15^N-glutamine labeling is shown quantitatively in the dot plot, where BrdU^+^ cells are red. Univariate comparisons between BrdU^−^ (blue) and BrdU^+^ (red) cells are shown by the box and whisker plots embedded in the graphs: ∗∗p < 0.0001, Mann-Whitney test. Note: y axis log_2_-scale. Representative of two tumors similarly labeled. Scale bar, 20 μm. (E) Dot plots demonstrating the dispersion of MIMS ^2^H-glucose measurements for different degrees of resolution. (F) Dot plots demonstrating the dispersion of MIMS ^15^N-glutamine measurements for different degrees of resolution. For (E) and (F): each “Tissue” data point is from a region of interest (ROI) encompassing an entire mosaic image and representing analyses of different tumor regions; “50 μm” indicates data points for 50 μm × 50 μm imaging fields and therefore each data point is inclusive of a cluster of cells; “Single cell” indicates the distributions where each data point is a single cell. Lines super-imposed on dot plots = median. See also Figure S4.

In order to assess the degree of tumor metabolic heterogeneity *in vivo*, we utilized MIMS to track labeled metabolic substrates, using a cocktail consisting of ^2^H-glucose, ^15^N-glutamine, and BrdU administered over 24 h to murine tumor models (Figure 2A). Our choice of label dose reflected our goal to achieve sufficiently high labeling in order to facilitate label detection within a reasonable amount of time and prior experience with amino acid and glucose metabolic labeling in other physiological or pathophysiological contexts (Guillermier et al., 2017b, 2019; Zhang et al., 2012). We first labeled an *Nf1*^*+/−*^*;Tp53*^*+/−*^ mouse model of malignant peripheral nerve sheath tumor (MPNST), incidentally finding a second intra-abdominal histiosarcoma, which in both tumor types is caused by a stochastic loss of wild-type *Nf1* and *Tp53* alleles. These tumors revealed intra- and inter-tumor heterogeneity in both glucose and glutamine labeling, evident in the MIMS images and when the underlying quantitative data was plotted (Figure 2B). Similar to cultured cancer cells (Figures 1C and 1D), there was a significant upward shift in label incorporation by the population of divided (BrdU^+^) cells in both tumors, although the distributions overlapped (Figure 2B). Importantly, the MPNST and histiosarcoma tumors arose in a genetically engineered mouse model and therefore may share similar driver mutations; however, the labeling patterns were distinct. The glucose and glutamine signals were generally correlated in the MPNST (R^2^ = 0.33, p < 0.0001), whereas such a relationship was not evident in the histiosarcoma, in part due to a subset of cells with high glucose labeling and markedly less glutamine labeling (Figure 2C). In a melanoma xenograft model (Figure 2D), we also observed heterogeneity in label uptake, again finding an increase in the labeling distribution of the BrdU^+^ population and a correlation between glucose and glutamine labeling (R^2^ = 0.45, p < 0.0001), similar to the MPNST model. When we compared the heterogeneity of the tumors relative to the cell lines using the metric of dispersion (MAD), we found a generally higher degree of heterogeneity in the tumors relative to the cultured cancer cell lines, although this was primarily driven by heterogeneity of glucose labeling (Figure S4).

We next considered the degree to which characterization of heterogeneity was dependent on a method with single cell resolution. We quantified the isotope ratio and therefore the degree of labeling revealed by MIMS analyses at different degrees of resolution. In order to accomplish this, we compared data for single cell regions of interest (ROIs), relative to data from ROIs that contained progressively larger analytical territories, thereby simulating lower spatial resolution (Figures 2E and 2F). This analysis demonstrated that increasing spatial resolution to capture individual cells with MIMS results in a broader distribution and an increase in the metric of dispersion (MAD). It also underscores what is visually evident in the MIMS images (e.g., Figure 2D), that variability in glucose and glutamine utilization was evident even in immediately adjacent cells occupying a similar tumor environment. Collectively, these initial MIMS data in three different tumor types demonstrate (1) augmentation of glucose and glutamine utilization by proliferating tumor cells *in vivo* and (2) a degree of heterogeneity that would not have been revealed with tissue-scale methods.

### MIMS Signature of Metabolic Heterogeneity Is Associated with Tumor Cell Proliferation

**Figure 3:**
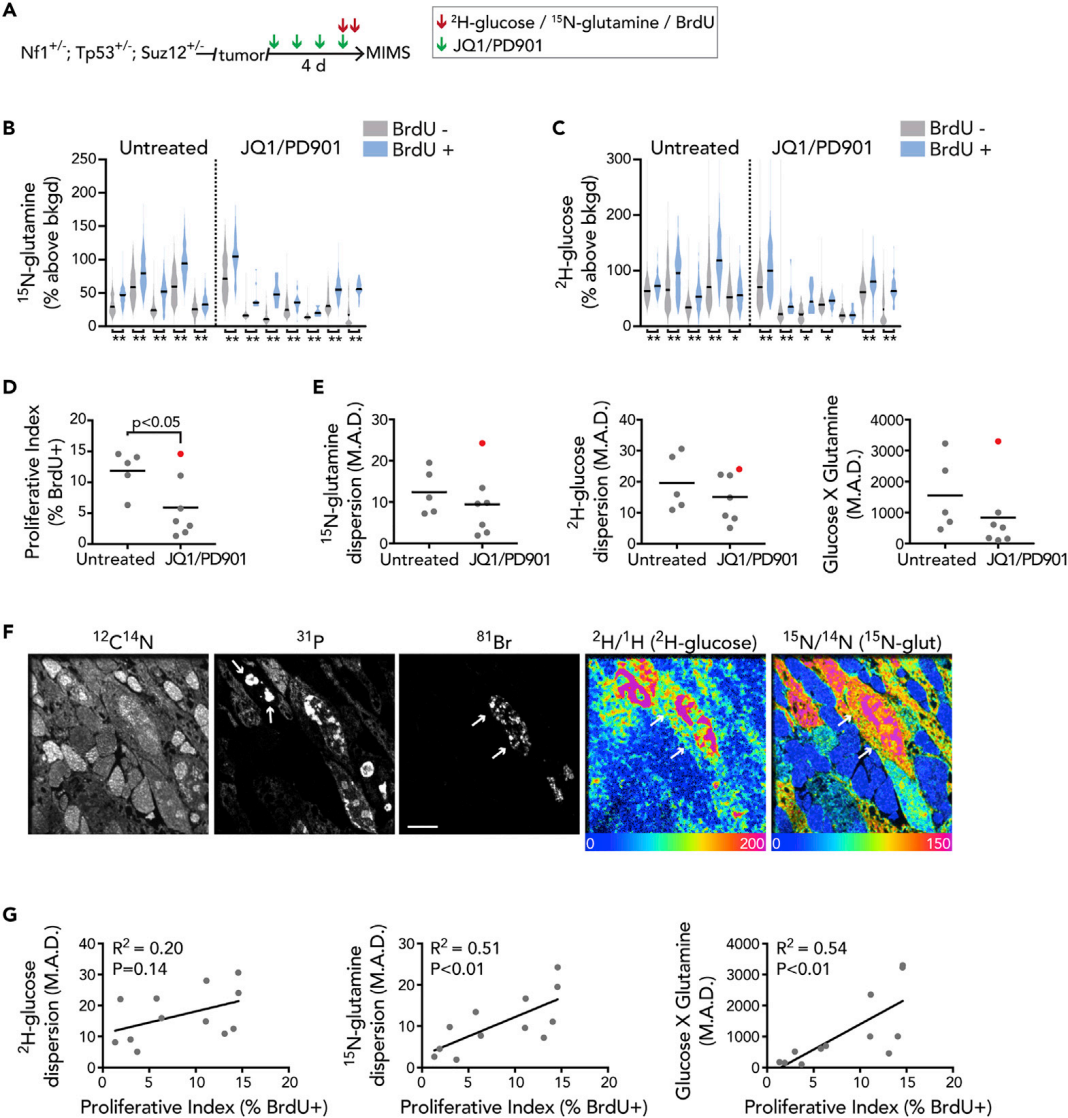
MIMS Signature of Metabolic Heterogeneity Is Associated with Tumor Growth (A) Experimental schematic. Tumor bearing *Nf1*^*+/−*^*; Tp53*^*+/−*^*; Suz12*^*+/−*^ mice were treated with a molecularly targeted therapy consisting of a bromodomain inhibitor (JQ1: 45 mg/kg/day) and an MEK inhibitor (PD901: 1.5 mg/kg/day). Therapy consisted of four daily doses. ^2^H-glucose, ^15^N-glutamine, and bromodeoxyuridine (BrdU) were administered in the 24 h prior to sacrifice as in Figure 2. (B) ^15^N-glutamine labeling of the BrdU^−^ (gray) and BrdU^+^ (blue) populations is shown by the violin plots for each tumor analyzed in the untreated and JQ1/PD901-treated groups. (C) ^2^H-glucose labeling of the BrdU^−^ (gray) and BrdU^+^ (blue) populations are shown by the violin plots for each tumor analyzed in the untreated and JQ1/PD901 treated groups. For (B) and (C), violin plot line = median ∗p < 0.05, ∗∗p < 0.001, Mann-Whitney tests. (D) Frequency of BrdU-labeled cells (proliferative index) in treated versus untreated tumors. Significance assessed by two-tailed t test. (E) Median absolute deviation (MAD) as a metric of dispersion/heterogeneity for ^2^H-glucose (left), ^15^N-glutamine (middle), and the product of the two labels (right). The tumor with the highest metric of dispersion (red dot) was also the tumor with the highest proliferative index in (D). (F) Images of representative region of treated tumor with the highest proliferative index and highest metric of dispersion (red dot) reveals bands of rounded cells either lacking the typical phosphorus signal emanating from chromatin or with a condensed signal consistent with a pyknotic nucleus. Between these dead cells are bands of heterogeneously labeled cells, many of which were BrdU^+^, consistent with proliferation through therapy. Scale bar, 5 μm. (G) Correlations between the degree of heterogeneity (MAD) and the proliferative index for the respective tumor. Similar findings were noted when the analyses were restricted to the proliferating (BrdU^+^) subpopulation (Figure S6). See also Figures S5–S7, Table S1.

Having demonstrated heterogeneity of intratumor substrate utilization, we considered its functional relevance. We turned our attention to a validated therapeutic model: the *Nf1*^*+/−*^*/Tp53*^*+/−*^*/Suz12*^*+/−*^ model of MPNST, which responds to a molecularly targeted combination of an MEK inhibitor (PD901) and a bromodomain inhibitor (JQ1) (De Raedt et al., 2014). We administered JQ1/PD901 for 4 days and labeled the mice in the final day prior to sacrifice in order to test the hypothesis that therapy would select for metabolically distinct subpopulations of tumor cells, thereby compressing heterogeneity (Figure 3A). We also reasoned that this larger cohort of tumors in a different genetic model would enable assessment of the consistency of our initial observations of metabolic heterogeneity. The tumors in these mice displayed a heterogeneous labeling pattern that was similar to data shown in Figure 2 and that was particularly striking when contrasted to the low level of labeling in peripheral nerves, which contain the cell of origin (Figures S5 and S6). All of the analyzed tumors, both treated and untreated, displayed a significant correlation between ^2^H-glucose and ^15^N-glutamine labeling (Table S1), similar to what was observed in previously imaged tumors (Figure 2). All of the tumors also displayed a significant upward shift in the ^15^N-glutamine labeling distributions in the subpopulation of BrdU^+^ cells (Figure 3B), and most of the tumors displayed a significant upward shift in the ^2^H-glucose labeling distribution in the BrdU^+^ cells (Figure 3C). The general increase in labeling in the fraction of BrdU^+^ cells suggested that a component of the observed heterogeneity was due to tumor cell proliferation. In order to assess this, we compared the metric of dispersion (MAD) for each tumor, with and without inclusion of the BrdU^+^ subpopulations (Table 1). When the BrdU^+^ subpopulations were excluded, the resultant metabolic labeling dispersions ranged from 78% to 100% of the MAD for the total population. One caveat to consider with analyses restricted to the BrdU-negative population is the degree to which BrdU labeling captures cycling cells. The BrdU analysis captures cells that have replicated their genome during S-phase in the 24 h preceding sacrifice of the mouse. We cannot exclude the possibility that a fraction of highly metabolically labeled, yet BrdU^−^ cells, have been captured in the process of entering S-phase, in essence an anticipatory augmentation of anabolic processes. Nonetheless, these collective data suggest that, although the population of cells that have replicated their genome and other components of biomass as part of cell division do tend to increase statistical dispersion of metabolic labeling, tumor metabolic heterogeneity is present independent of cell division.

**Table 1:** Contribution of the Proliferated Population to MIMS Assessment of Heterogeneity in Malignant Peripheral Nerve Sheath Tumors

	Glutamine			Glucose			Glutamine X Glucose		
Tumor	MAD	MAD (BrdU^−^)	% Total	MAD	MAD (BrdU^−^)	% Total	MAD	MAD (BrdU^−^)	% Total

**Untreated tumors**									

1	11.1	9.5	85.7	12.5	12.6	101.0	1,003.8	893.9	89.1
2	16.7	16.6	99.0	28.0	26.7	95.3	2,353.9	2,194.8	93.2
3	7.2	5.9	81.2	10.9	10.0	92.0	459.6	384.1	83.6
4	19.5	17.5	89.4	30.6	26.1	85.4	3,232.0	2,621.3	81.1
5	7.7	7.7	100.2	15.9	16.0	100.6	698.7	692.1	99.1

**JQ1/PD901 treated**									

1	24.3	22.6	92.9	24.1	22.8	94.9	3,300.3	2,819.0	85.4
2	13.4	12.2	91.1	22.3	20.2	90.5	607.1	476.1	78.4∗
3	2.6	2.6	98.1	8.1	8.0	98.9	174.7	171.8	98.3
4	4.5	4.3	94.4	10.1	9.9	98.5	152.0	147.9	97.3
5	9.8	7.9	80.6	9.0	8.4	92.5	516.5	446.4	86.4
6	1.9	1.8	95.7	5.1	5.1	100.0	102.4	100.0	97.7
7	9.6	7.9	82.1	14.9	14.1	94.6	1002.9	872.8	87.0

Removal of the proliferated (BrdU^+^) cells from the analyses resulted in a reduction in the metric of dispersion (MAD) that was modest (∗max = 21.6% absolute reduction).

We next assessed for a treatment effect. Consistent with previous observations, the majority of these tumors began to shrink and the proliferative index of remaining cells—as indicated by the frequency of BrdU^+^ cells—was reduced (Figure 3D). There was a nonsignificant reduction in heterogeneity as indicated by the median absolute deviation in both glucose and glutamine labeling (Figure 3E). Notably, the treated tumor with the greatest heterogeneity was the tumor with the highest proliferative index after drug treatment, thus representing a putative resistant tumor (Figure 3F and indicated by red dot in Figures 3D and 3E). When we examined the labeling distributions, we discovered a positive correlation between heterogeneity and the proliferative index (Figure 3G). This relationship was also evident if we restricted the analysis to the subpopulation of BrdU^+^ cells (Figure S7), suggesting that the augmented heterogeneity in the highly proliferative tumors was not simply a manifestation of the upward shift and expansion of the labeling distribution due to the proliferative BrdU^+^ cells. Contrary to our initial hypothesis, these data suggest that the fastest growing and/or resistant tumors are not characterized by selection for a dominant metabolically distinct tumor cell population; instead, the degree of metabolic heterogeneity is predictive of tumor cell proliferation.

### MIMS Signature of Metabolic Heterogeneity Is Not Generalizable to Non-malignant Cells

**Figure 4:**
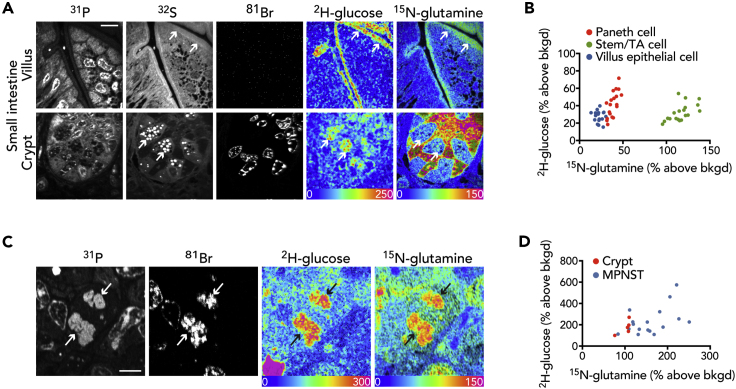
MIMS Signature of Metabolic Heterogeneity Is Not Generalizable to Non-malignant Cells (A) Representative images of small intestine. Top: villus lined by terminally differentiated, non-dividing (BrdU^−^) epithelial cells and demonstrating a uniform stripe of labeling corresponding to the brush border (arrows). Bottom: Paneth cells identifiable by their stereotypical sulfur-rich granules (arrows) are surrounded by proliferating cells (BrdU^+^) that are stem/transit amplifying cells. Scale bar, 10 μm. (B) Dot plot of cells from (E). Individual cell types (color coded) segregate by labeling intensity. Note: only the stem/TA cells are proliferative (BrdU^+^). TA, transit amplifying cell. (C) Mitotic figure from MPNST. Segregating chromosomes (arrows), evident in the ^31^P images, are also BrdU labeled. Scale bar, 5 μm. (D) Dot plot demonstrating heterogeneity of glutamine and glucose labeling of MPNST mitotic figures relative to mitotic figures from the small intestinal crypt as also indicated by the median absolute deviation: MPNST glucose (MAD = 54.8) and glutamine (MAD = 35.5) versus crypt glucose (MAD = 36.3) and glutamine (MAD = 1.6).

In order to investigate whether heterogeneity in glucose and glutamine labeling is a more generalizable feature of proliferating cells, we examined the small intestine (Figure 4A), which physiologically replaces its epithelial cell layer every 3–5 days (Steinhauser et al., 2012). Paneth cells, which are terminally differentiated secretory cells in the crypt, demonstrated intense labeling in their stereotypical granules. The terminally differentiated villus epithelial cells demonstrated a homogeneously intense labeling pattern at the metabolically active brush border. Given that neither of these terminally differentiated cell populations divide, it is perhaps not surprising that they would exhibit a more organized labeling pattern. However, we also examined the stem and transit amplifying cells at the base of the small intestinal crypt, which proliferate under homeostatic conditions and therefore become uniformly BrdU labeled. In contrast to the heterogeneity observed in proliferating tumor cells, the proliferating cells in the crypt segregated into a discrete population based on glucose and glutamine labeling (Figure 4B). As expected, these data confirm that physiological cell populations also utilize metabolic substrate for anabolism; however, the process is uniform relative to the pathological cancer state.

Since glucose and glutamine may serve as substrate for both protein and nucleic acid synthesis, we reasoned that analysis of newly synthesized chromosomes in MPNST mitotic figures would provide insight into these biosynthetic pathways (Figure 4C). The proliferative activity in the small intestinal crypt also results in histologically visible mitotic figures, thereby providing a physiological control relative to pathologic cancer proliferation (Steinhauser et al., 2012). Although labeling was detectable in the segregating chromosomes in both contexts, the glucose and glutamine signal was more heterogeneous in the mitotic cancer cells as indicated by an increased metric of dispersion (median absolute deviation, MAD, Figure 4D), suggesting that the signal of tumor metabolic heterogeneity extends to the replication of chromosomes.

### Mosaic Yap Transgenesis in the Liver Reveals Cell Autonomous and Non-cell Autonomous Metabolic Reprogramming

**Figure 5:**
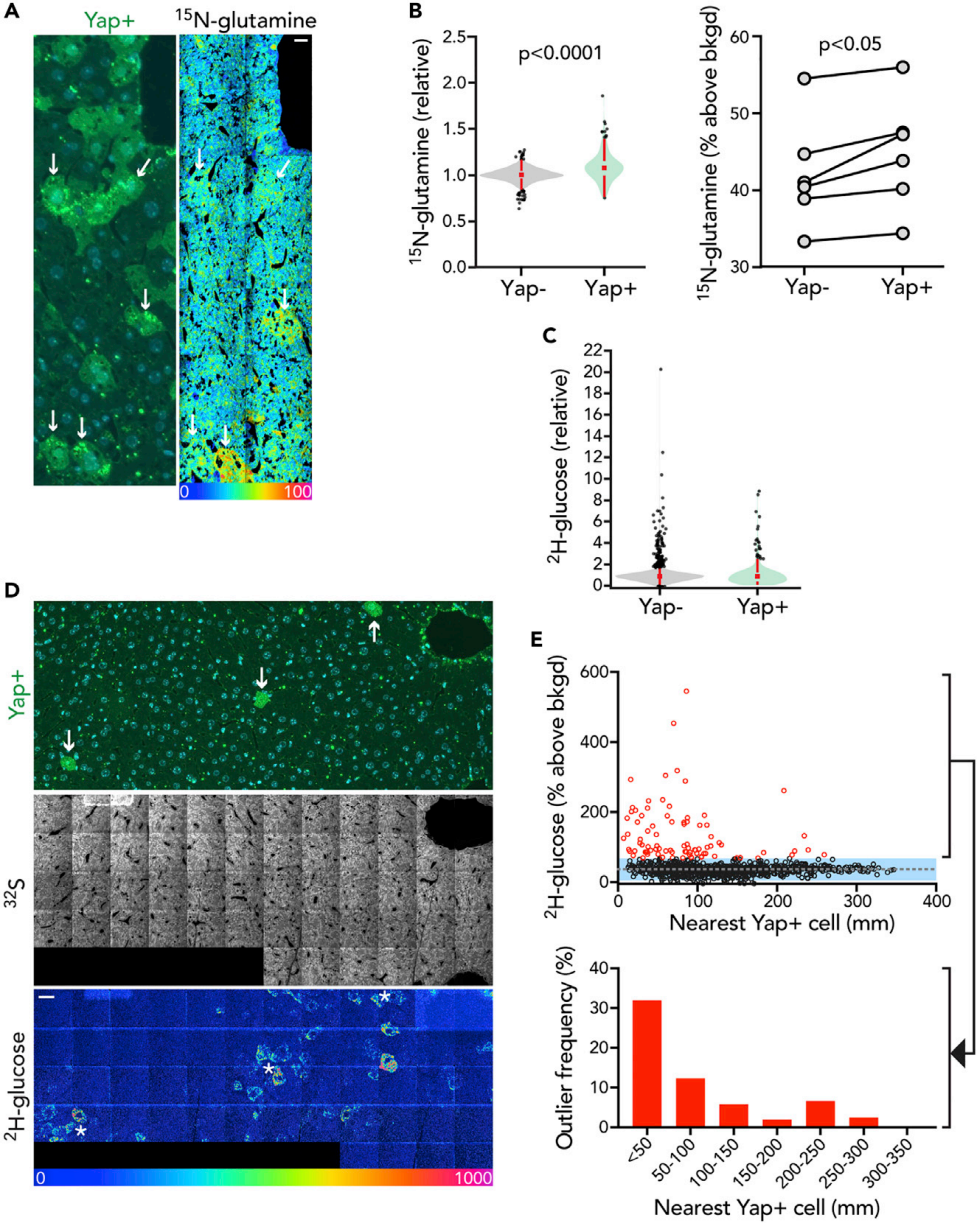
Mosaic Yap Transgenesis in the Liver Reveals Cell Autonomous and Non-cell Autonomous Metabolic Reprogramming Mosaic Yap overexpression achieved by administering a hepatocyte-specific AAV-Cre to facilitate doxycycline-inducible YAP expression. Mice were labeled with ^2^H-glucose and ^15^N-glutamine 10 days later. (A) Left: Immunofluorescence staining of the AAV-reporter (tdTomato). Right: adjacent mosaic HSI image of ^15^N-glutamine (^12^C^15^N/^12^C^14^N) labeling. Arrows: select Yap+ cells reveal visibly higher glutamine labeling. Scale bar, 10 μm. (B) Left: distribution of ^15^N-glutamine labeling in wild-type and Yap+ cells. Pooled analysis of all analyzed cells with significance assessed by Mann-Whitney test. Right: mean ^15^N-glutamine signal in Yap+ versus wild-type hepatocytes within each mouse (n = 6, two-tailed, paired t test). (C) Distribution of ^2^H-glucose labeling in wild-type and Yap+ cells. Pooled analysis of all analyzed cells. (D) Top: Representative region with scant Yap+ cells (arrows). Bottom: mosaic HSI image demonstrates clusters of intense ^2^H-glucose labeled cells in the vicinity of Yap+ cells (asterisks). Scale bar, 20 μm. (E) Top: Plot of ^2^H-glucose labeling as a function of linear proximity to the nearest identifiable Yap+ cell. Glucose-avid cells, defined as those exceeding 2 SD (blue shade) from the mean (dotted line) are shown in red; their frequency as a function of distance from Yap+ cell is shown in the histogram on the bottom. See also Figures S8 and S9.

One question raised by these data is the degree to which heterogeneity of glucose and glutamine utilization by tumors reflects individual cell autonomous genetic programs. In a melanoma xenograft, for example, we observed rare clusters of similarly glucose-avid cells suggestive of possible metabolically distinct clones (Figure S8). In the same tumor, we also observed augmentation of substrate utilization in the core of the tumor relative to the margin. Although we did not observe such visually dramatic clustering beyond this melanoma, we also considered the possibility that the MPNST tumor cohort might exhibit more subtle clustering. To test this, we selected highly labeled outlier cells and examined the labeling distributions of adjacent cells relative to randomly selected cell clusters distant to the outliers. Although lower than the outliers themselves, cells adjacent to highly labeled outliers demonstrated a modest, yet significant, augmentation in glucose and glutamine labeling relative to more distant control populations (Glutamine labeling median = 39.9% versus control = 33.1% above background, p < 0.05; Glucose labeling median = 79.9% versus control 50.1% above background, p < 0.0001). This observation suggests metabolic clustering, albeit to a more subtle degree than observed in the melanoma. Such clustering could implicate a metabolic effect related to the microenvironment, such as differences in oxygen tension or pH, or alternatively could arise as a consequence of a genetic subclone (Figure S8). Therefore, as proof of concept, we tested whether targeted activation of a relevant oncogenic pathway, first in normal tissue, would modulate glucose or glutamine avidity in a cell autonomous manner. We selected the Yap pathway for overexpression because it is frequently activated in diverse cancers and it is also known to promote utilization of glucose and glutamine for anabolic growth (Cox et al., 2016, 2018; Zanconato et al., 2016). We delivered a hepatocyte-specific Cre-recombinase to activate expression of a doxycycline-inducible Yap transgene as previously reported (Yimlamai et al., 2014). This resulted in mosaic Yap activation in the liver as marked by a tdTomato reporter (Figure 5A). Yap overexpressing hepatocytes augmented ^15^N-glutamine labeling relative to control non-transfected cells that was subtle but visually appreciable (Figure 5A) and quantitatively evident as an increase in the corresponding ^15^N-labeling distribution (Figure 5B). We also observed a subtle negative gradient of ^15^N-glutamine labeling from extending from the portal triad toward the more hypoxic environment of the central vein (Figure S9). In contrast to glutamine, the distribution of ^2^H-glucose labeling in both Yap^+^ and Yap^−^ hepatocytes was more heterogeneous; even when we performed a pooled comparison, we detected no significant difference between the two cell populations (Figure 5C). In regions of lowest effective Yap transgenesis where there were sufficiently broad swathes of untransfected control hepatocytes, however, we observed untransfected glucose avid cells clustering with Yap^+^ cells (Figure 5D). Specifically, the frequency of glucose-avid outliers—defined as ^2^H-glucose signal 2σ from the mean—increased as a function of proximity to a Yap^+^ cell (Figure 5E). Therefore, these data are consistent with the concept that overexpression of an oncogenic pathway can modulate metabolism both by cell autonomous mechanisms and also through effects on neighboring cells, perhaps via paracrine signals.

## Discussion

In summary, we have quantified ^2^H-glucose and ^15^N-glutamine labeling, together with a label for cell division, at subcellular resolution in cancer cell lines and murine tumors with MIMS. In cancer cell lines and murine tumor models, proliferating cells demonstrated augmented glucose and glutamine utilization, consistent with the concept that cancer cells avidly utilize both substrates to support biomass (DeBerardinis et al., 2007; Vander Heiden and DeBerardinis, 2017). Importantly, we also observed intense metabolic labeling in non-cancerous, proliferating stem and transit amplifying cells of the small intestinal crypt in mice. Indeed, it was not the absolute degree of metabolic labeling but its heterogeneity that was most evident in tumors.

That cancer cells augment utilization of anabolic substrates such as glucose and glutamine to support proliferation and growth is not new, nor is the concept that tumors exhibit metabolic heterogeneity. Metabolic imaging studies at tissue-scale resolution and metabolic flux analyses of tumor samples have revealed heterogeneity of a variety of metabolic parameters (Hensley et al., 2016; Salamanca-Cardona et al., 2017; Sengupta and Pratx, 2016). However, this study affirms and/or extends prior findings in at least three important ways, in demonstrating (1) increased substrate utilization by individual proliferating cancer cells *in vivo*; (2) metabolic heterogeneity of individual cancer cells, independent of factors that may confound tissue-scale analyses such as regional variability in viability (e.g., necrosis) or proliferation; and (3) that heterogeneity plays out within small clusters of adjacent cells exposed to similar tumor environments. Collectively, the degree of heterogeneity demonstrated by MIMS would not have been appreciated with lower resolution methods operating at tissue scale.

What is the significance of the heterogeneity of MIMS metabolic labeling measurements? Our *a priori* hypothesis was that metabolic heterogeneity would be analogous to genetic heterogeneity: that subpopulations of metabolically distinct tumor cells would be programmed for survival and proliferation and therefore the emergence of resistant cells would manifest as relatively homogeneous metabolic clones. Instead, we observed in an MPNST model that heterogeneity of substrate utilization persisted in proliferating cells that escaped targeted therapy. In addition, the degree of heterogeneity correlated with tumor cell proliferation. We speculate that the MIMS signal of heterogeneity reflects an underlying metabolic flexibility that promotes survival, proliferation, and/or treatment resistance.

Diverse cellular mechanisms may account for the heterogeneity observed in this study. We first considered the degree to which heterogeneity was driven by increased metabolic labeling due to subpopulations of dividing cells. Although the metric of heterogeneity used in this study (MAD) generally decreased when we excluded the population of divided cells from the analysis, the reduction was generally modest, reflecting substantial overlap in the labeling distributions of the divided (BrdU^+^) and undivided cell populations. We also considered the plausibility that heterogeneity of MIMS signal could arise due to underlying genetic heterogeneity. In the majority of MIMS images, we observed a pattern of chaotic cell-to-cell variability in labeling, with limited examples of anatomically localized and metabolically distinct clusters, which could be indicative of a clonal population. In addition, when we directly examined the effect of manipulating a single oncogenic gene by transgenic activation of the Yap pathway in otherwise normal liver, we found a pattern of heterogeneity consistent with both cell autonomous and non-autonomous effects. This result in normal liver provides context to the cancer setting where molecular derangements are compounded and superimposed on variable cell cycle states, suggesting that the MIMS signal likely reflects a complex interplay between each cell's own molecular program, its cell cycle state, and microenvironmental pressures.

An important question raised by our study is the degree to which metabolic heterogeneity extends to other tumor types and to human cancer. A potential criticism of mouse tumor models—both those driven by genetically engineered driver mutations and by human xenografts where the transplantation of cancer cells may select for proliferative fitness—is that they may be more homogeneous than spontaneous human cancers (Alizadeh et al., 2015). The extensive precedent of human stable isotope studies and prior successful translation of MIMS to studies of human cell turnover suggests that this translational question will likely be addressable, particularly if technical challenges including efficiency of sample analysis and automation of image analysis and data processing are resolved (Guillermier et al., 2017a; Steinhauser et al., 2012; Steinhauser and Lechene, 2013). In the current study, we mitigated the throughput limitations of MIMS in part by imaging at less than the maximal lateral imaging resolution (approximately 30 nm). As such, we did not perform analyses at the degree of resolution necessary to resolve organelles beyond the largest structures, such as nuclei. In light of recent work demonstrating heterogeneity of protein turnover in individual lysosomes, averaging the MIMS signal over the entire cross-section of each cell did not directly account for variability in substrate utilization by individual organelles or other intracellular structures (Narendra et al., 2020). In addition, although the precise measurement of stable isotope tracers at high spatial resolution is unparalleled, MIMS does not provide molecular specificity beyond what is conferred by the parent tracer molecule. Therefore, our study may still underestimate the degree of metabolic heterogeneity exhibited by spontaneously arising human tumors, which may be revealed in greater mechanistic detail by future integration of MIMS measurements with complementary flux analyses of pooled cells as achievable with other mass spectrometry methods (Hensley et al., 2016), genetic or antibody-based reporters of protein markers (Keren et al., 2018), and low-input genomics analyses (Janiszewska et al., 2015; Patel et al., 2014). In establishing tumor metabolic heterogeneity and its functional significance for individual tumor cells, MIMS holds promise to inform the development of therapies that target tumor metabolism.

### Limitations of the Study

This study reflects limitations inherent to the multi-isotope imaging mass spectrometry platform (NanoSIMS) used to perform metabolic tracer measurements. Analytical throughput remains a limitation, as indicated by the modest number of tumors analyzed in this study. Optimization of analytical strategies will be required in order to render larger studies and human translational studies more practical. A second issue relates to the lack of molecular specificity, since isotope ratio measurements track heavy atoms, not specific metabolites. Given diverse potential metabolic fates of ^2^H-glucose and ^15^N-glutamine labels, intracellular signals indicate substrate utilization via different metabolic pathways.

### Resource Availability

#### Lead Contact

Further information and requests for resources and protocols should be directed to the Lead Contact, Matthew Steinhauser (msteinhauser@pitt.edu).

#### Materials Availability

This study did not generate new unique reagents.

#### Data and Code Availability

Datasets are available upon request.

## Methods

All methods can be found in the accompanying Transparent Methods supplemental file.

## Supplemental Information

Document S1. Transparent Methods, Figures S1–S9, and Table S1

## Footnotes

Supplemental Information can be found online at https://doi.org/10.1016/j.isci.2020.101355.

## References

[bib1] Alizadeh A.A., Aranda V., Bardelli A., Blanpain C., Bock C., Borowski C., Caldas C., Califano A., Doherty M., Elsner M. (2015). Toward understanding and exploiting tumor heterogeneity. Nat. Med..

[bib2] Anderson K., Lutz C., van Delft F.W., Bateman C.M., Guo Y., Colman S.M., Kempski H., Moorman A.V., Titley I., Swansbury J. (2011). Genetic variegation of clonal architecture and propagating cells in leukaemia. Nature.

[bib3] Azizi E., Carr A.J., Plitas G., Cornish A.E., Konopacki C., Prabhakaran S., Nainys J., Wu K., Kiseliovas V., Setty M. (2018). Single-cell map of diverse immune phenotypes in the breast tumor microenvironment. Cell.

[bib4] Brooks F.J. (2013). On some misconceptions about tumor heterogeneity quantification. Eur. J. Nucl. Med. Mol. Imag..

[bib5] Coimbra A., Leblond C.P. (1966). Sites of glycogen synthesis in rat liver cells as shown by electron microscope radioautography after administration of glucose-H3. J. Cell Biol..

[bib6] Cox A.G., Hwang K.L., Brown K.K., Evason K., Beltz S., Tsomides A., O'Connor K., Galli G.G., Yimlamai D., Chhangawala S. (2016). Yap reprograms glutamine metabolism to increase nucleotide biosynthesis and enable liver growth. Nat. Cell Biol..

[bib7] Cox A.G., Tsomides A., Yimlamai D., Hwang K.L., Miesfeld J., Galli G.G., Fowl B.H., Fort M., Ma K.Y., Sullivan M.R. (2018). Yap regulates glucose utilization and sustains nucleotide synthesis to enable organ growth. EMBO J..

[bib8] De Feyter H.M., Behar K.L., Corbin Z.A., Fulbright R.K., Brown P.B., McIntyre S., Nixon T.W., Rothman D.L., de Graaf R.A. (2018). Deuterium metabolic imaging (DMI) for MRI-based 3D mapping of metabolism in vivo. Sci. Adv..

[bib9] De Raedt T., Beert E., Pasmant E., Luscan A., Brems H., Ortonne N., Helin K., Hornick J.L., Mautner V., Kehrer-Sawatzki H. (2014). PRC2 loss amplifies Ras-driven transcription and confers sensitivity to BRD4-based therapies. Nature.

[bib10] DeBerardinis R.J., Mancuso A., Daikhin E., Nissim I., Yudkoff M., Wehrli S., Thompson C.B. (2007). Beyond aerobic glycolysis: transformed cells can engage in glutamine metabolism that exceeds the requirement for protein and nucleotide synthesis. Proc. Natl. Acad. Sci. U S A.

[bib11] Eary J.F., O'Sullivan F., O'Sullivan J., Conrad E.U. (2008). Spatial heterogeneity in sarcoma 18F-FDG uptake as a predictor of patient outcome. J. Nucl. Med..

[bib12] Fan T.W., Lane A.N., Higashi R.M., Farag M.A., Gao H., Bousamra M., Miller D.M. (2009). Altered regulation of metabolic pathways in human lung cancer discerned by (13)C stable isotope-resolved metabolomics (SIRM). Mol. Cancer.

[bib13] Faubert B., Li K.Y., Cai L., Hensley C.T., Kim J., Zacharias L.G., Yang C., Do Q.N., Doucette S., Burguete D. (2017). Lactate metabolism in human lung tumors. Cell.

[bib14] Fletcher J.W., Djulbegovic B., Soares H.P., Siegel B.A., Lowe V.J., Lyman G.H., Coleman R.E., Wahl R., Paschold J.C., Avril N. (2008). Recommendations on the use of 18F-FDG PET in oncology. J. Nucl. Med..

[bib15] Guillermier C., Doherty S.P., Whitney A.G., Babaev V.R., Linton M.F., Steinhauser M.L., Brown J.D. (2019). Imaging mass spectrometry reveals heterogeneity of proliferation and metabolism in atherosclerosis. JCI Insight.

[bib16] Guillermier C., Fazeli P.K., Kim S., Lun M., Zuflacht J.P., Milian J., Lee H., Francois-Saint-Cyr H., Horreard F., Larson D. (2017). Imaging mass spectrometry demonstrates age-related decline in human adipose plasticity. JCI Insight.

[bib17] Guillermier C., Poczatek J.C., Taylor W.R., Steinhauser M.L. (2017). Quantitative imaging of deuterated metabolic tracers in biological tissues with nanoscale secondary ion mass spectrometry. Int. J. Mass Spectrom..

[bib18] Gyngard F., Steinhauser M.L. (2019). Biological explorations with nanoscale secondary ion mass spectrometry. J. Anal. At. Spectrom..

[bib19] Hampel F.R. (1974). The influence curve and its role in robust estimation. J. Am. Stat. Assoc..

[bib20] Hensley C.T., Faubert B., Yuan Q., Lev-Cohain N., Jin E., Kim J., Jiang L., Ko B., Skelton R., Loudat L. (2016). Metabolic heterogeneity in human lung tumors. Cell.

[bib21] Hu F., Chen Z., Zhang L., Shen Y., Wei L., Min W. (2015). Vibrational imaging of glucose uptake activity in live cells and tissues by stimulated Raman scattering. Angew. Chem. Int. Ed. Engl..

[bib22] Jackson H.W., Fischer J.R., Zanotelli V.R.T., Ali H.R., Mechera R., Soysal S.D., Moch H., Muenst S., Varga Z., Weber W.P. (2020). The single-cell pathology landscape of breast cancer. Nature.

[bib23] Janiszewska M., Liu L., Almendro V., Kuang Y., Paweletz C., Sakr R.A., Weigelt B., Hanker A.B., Chandarlapaty S., King T.A. (2015). In situ single-cell analysis identifies heterogeneity for PIK3CA mutation and HER2 amplification in HER2-positive breast cancer. Nat. Genet..

[bib24] Juweid M.E., Cheson B.D. (2006). Positron-emission tomography and assessment of cancer therapy. N. Engl. J. Med..

[bib25] Kang S.R., Song H.C., Byun B.H., Oh J.R., Kim H.S., Hong S.P., Kwon S.Y., Chong A., Kim J., Cho S.G. (2014). Intratumoral metabolic heterogeneity for prediction of disease progression after concurrent chemoradiotherapy in patients with inoperable stage III non-small-cell lung cancer. Nucl. Med. Mol. Imag..

[bib26] Kelloff G.J., Hoffman J.M., Johnson B., Scher H.I., Siegel B.A., Cheng E.Y., Cheson B.D., O'Shaughnessy J., Guyton K.Z., Mankoff D.A. (2005). Progress and promise of FDG-PET imaging for cancer patient management and oncologic drug development. Clin. Cancer Res..

[bib27] Keren L., Bosse M., Marquez D., Angoshtari R., Jain S., Varma S., Yang S.R., Kurian A., Van Valen D., West R. (2018). A structured tumor-immune microenvironment in triple negative breast cancer revealed by multiplexed ion beam imaging. Cell.

[bib28] Kidd E.A., Grigsby P.W. (2008). Intratumoral metabolic heterogeneity of cervical cancer. Clin. Cancer Res..

[bib29] Kim S.M., Lun M., Wang M., Senyo S.E., Guillermier C., Patwari P., Steinhauser M.L. (2014). Loss of white adipose hyperplastic potential is associated with enhanced susceptibility to insulin resistance. Cell Metab..

[bib30] Lechene C., Hillion F., McMahon G., Benson D., Kleinfeld A.M., Kampf J.P., Distel D., Luyten Y., Bonventre J., Hentschel D. (2006). High-resolution quantitative imaging of mammalian and bacterial cells using stable isotope mass spectrometry. J. Biol..

[bib31] Maher E.A., Marin-Valencia I., Bachoo R.M., Mashimo T., Raisanen J., Hatanpaa K.J., Jindal A., Jeffrey F.M., Choi C., Madden C. (2012). Metabolism of [U-13 C]glucose in human brain tumors in vivo. NMR Biomed..

[bib32] Martin R.D., Zamar R.H. (1989). Asymptotically min-max bias robust M-estimates of scale for positive random variables. J. Am. Stat. Assoc..

[bib33] Narendra D.P., Guillermier C., Gyngard F., Huang X., Ward M.E., Steinhauser M.L. (2020). Coupling APEX labeling to imaging mass spectrometry of single organelles reveals heterogeneity in lysosomal protein turnover. J. Cell Biol..

[bib34] O'Neil R.G., Wu L., Mullani N. (2005). Uptake of a fluorescent deoxyglucose analog (2-NBDG) in tumor cells. Mol. Imag. Biol..

[bib35] Patel A.P., Tirosh I., Trombetta J.J., Shalek A.K., Gillespie S.M., Wakimoto H., Cahill D.P., Nahed B.V., Curry W.T., Martuza R.L. (2014). Single-cell RNA-seq highlights intratumoral heterogeneity in primary glioblastoma. Science.

[bib36] Salamanca-Cardona L., Shah H., Poot A.J., Correa F.M., Di Gialleonardo V., Lui H., Miloushev V.Z., Granlund K.L., Tee S.S., Cross J.R. (2017). In vivo imaging of glutamine metabolism to the oncometabolite 2-hydroxyglutarate in IDH1/2 mutant tumors. Cell Metab..

[bib37] Sengupta D., Pratx G. (2016). Imaging metabolic heterogeneity in cancer. Mol. Cancer.

[bib38] Steinhauser M.L., Bailey A.P., Senyo S.E., Guillermier C., Perlstein T.S., Gould A.P., Lee R.T., Lechene C.P. (2012). Multi-isotope imaging mass spectrometry quantifies stem cell division and metabolism. Nature.

[bib39] Steinhauser M.L., Lechene C.P. (2013). Quantitative imaging of subcellular metabolism with stable isotopes and multi-isotope imaging mass spectrometry. Semin. Cel. Dev. Biol..

[bib40] Tirosh I., Izar B., Prakadan S.M., Wadsworth M.H., Treacy D., Trombetta J.J., Rotem A., Rodman C., Lian C., Murphy G. (2016). Dissecting the multicellular ecosystem of metastatic melanoma by single-cell RNA-seq. Science.

[bib41] Torizuka T., Tamaki N., Inokuma T., Magata Y., Sasayama S., Yonekura Y., Tanaka A., Yamaoka Y., Yamamoto K., Konishi J. (1995). In vivo assessment of glucose metabolism in hepatocellular carcinoma with FDG-PET. J. Nucl. Med..

[bib42] Vander Heiden M.G., DeBerardinis R.J. (2017). Understanding the intersections between metabolism and cancer biology. Cell.

[bib43] Venneti S., Dunphy M.P., Zhang H., Pitter K.L., Zanzonico P., Campos C., Carlin S.D., La Rocca G., Lyashchenko S., Ploessl K. (2015). Glutamine-based PET imaging facilitates enhanced metabolic evaluation of gliomas in vivo. Sci. Transl. Med..

[bib44] Walsh A.J., Cook R.S., Sanders M.E., Aurisicchio L., Ciliberto G., Arteaga C.L., Skala M.C. (2014). Quantitative optical imaging of primary tumor organoid metabolism predicts drug response in breast cancer. Cancer Res..

[bib45] Weber W.A., Ziegler S.I., Thodtmann R., Hanauske A.R., Schwaiger M. (1999). Reproducibility of metabolic measurements in malignant tumors using FDG PET. J. Nucl. Med..

[bib46] Wei L., Yu Y., Shen Y., Wang M.C., Min W. (2013). Vibrational imaging of newly synthesized proteins in live cells by stimulated Raman scattering microscopy. Proc. Natl. Acad. Sci. U S A.

[bib47] Xu P., Oosterveer M.H., Stein S., Demagny H., Ryu D., Moullan N., Wang X., Can E., Zamboni N., Comment A. (2016). LRH-1-dependent programming of mitochondrial glutamine processing drives liver cancer. Genes Dev..

[bib48] Yimlamai D., Christodoulou C., Galli G.G., Yanger K., Pepe-Mooney B., Gurung B., Shrestha K., Cahan P., Stanger B.Z., Camargo F.D. (2014). Hippo pathway activity influences liver cell fate. Cell.

[bib49] Yoon H.J., Kim Y., Kim B.S. (2015). Intratumoral metabolic heterogeneity predicts invasive components in breast ductal carcinoma in situ. Eur. Radiol..

[bib50] Zanconato F., Cordenonsi M., Piccolo S. (2016). YAP/TAZ at the roots of cancer. Cancer Cell.

[bib51] Zhang D.S., Piazza V., Perrin B.J., Rzadzinska A.K., Poczatek J.C., Wang M., Prosser H.M., Ervasti J.M., Corey D.P., Lechene C.P. (2012). Multi-isotope imaging mass spectrometry reveals slow protein turnover in hair-cell stereocilia. Nature.

