# Supplementary material for: Imaging Mass Spectrometry Reveals Tumor Metabolic Heterogeneity
Source: iScience. 2020 Jul 10;23(8):101355. doi: 10.1016/j.isci.2020.101355 (PMC7390776; doi:10.1016/j.isci.2020.101355)
Supplement: Document S1. Transparent Methods, Figures S1–S9, and Table S1 [file mmc1.pdf]

iScience, Volume *23*

Supplemental Information

Imaging Mass Spectrometry Reveals

Tumor Metabolic Heterogeneity

Yang Zhang, Christelle Guillermier, Thomas De Raedt, Andrew G. Cox, Ophelia Maertens, Dean Yimlamai, Mingyue Lun, Adam Whitney, Richard L. Maas, Wolfram Goessling, Karen Cichowski, and Matthew L. Steinhauser

Supplemental Figures:

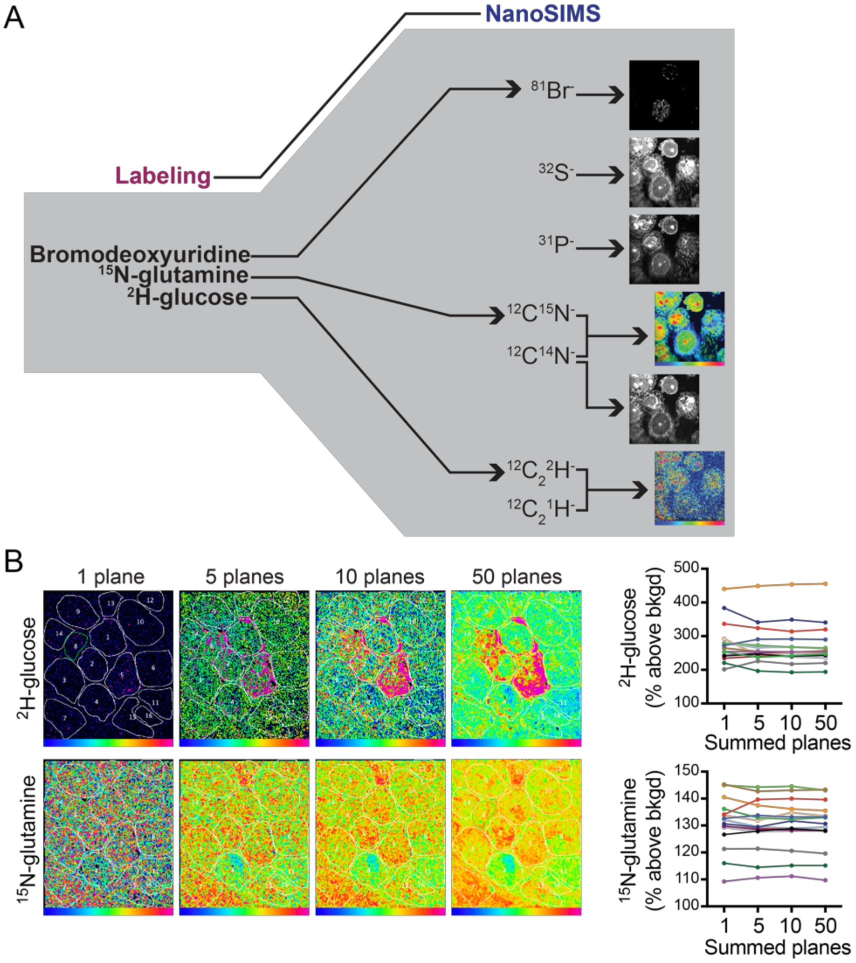

Figure S1. Application of multi-isotope imaging mass spectrometry (MIMS) to cancer, related to Figures 1-5. (A) Schematic depicting measurement of glucose and glutamine utilization. Stable isotope metabolic tracers and the cell cycle label, bromodeoxyuridine (BrdU), were administered to cultured cells or to tumor bearing mice. Histological sections (0.5 μm) are mounted on silicon wafers and analyzed with a NanoSIMS instrument, which rasters the sample with a primary Cs ion beam, sputtering the surface atomic layers. The ionized fraction is shaped by ion optics and transmitted to the mass spectrometer, where up to seven discrete ions are counted in parallel (7 detectors). Mass images of CN, P, and S reveal histological details. Direct measurement of Br reveals incorporation of BrdU. The parallel acquisition of rare and common isotopic variants enables quantification of isotope ratios and the generation of Hue Saturation Intensity (HSI) ratio images. Polyatomic clusters are often used for isotope ratio measurements. For example, due to the negligible electron affinity of N, the $^{15}N/^{14}N$ ratio is instead measured using $^{12}C^{15}N/^{12}C^{14}N$. **(B)** Each pixel represents a discrete measurement from a volume with lateral dimensions as low as 30nm and a depth of a few atomic layers. In an HSI image, pixels with low ion counts appear dark. In order to achieve sufficiently high statistical confidence for each pixel measurement, dwell time at each pixel can be increased and/or multiple planes acquired sequentially and summed. If successive planes of increasing depth are summed as shown from left to right, each pixel becomes more intense indicative of an improved counting statistic. This occurs more rapidly for $^{12}C^{15}N/^{12}C^{14}N$ due to the higher ion yield. In this study, however, data points were derived from regions of interest (ROIs, traced in white) corresponding to individual cells, merging ion counts for all of the pixels within an ROI. As a consequence, elevations in the isotope ratio are evident prior to the label being visually obvious in the HSI image (right) because the collective ion counts within an ROI provide a stronger statistical measure than individual pixels. The graphed data on the right correspond to the ROIs outlined in the images on the left demonstrate a reasonably accurate representation of the distribution of isotope ratio measurements for the selected ROIs after just one analytical plane.

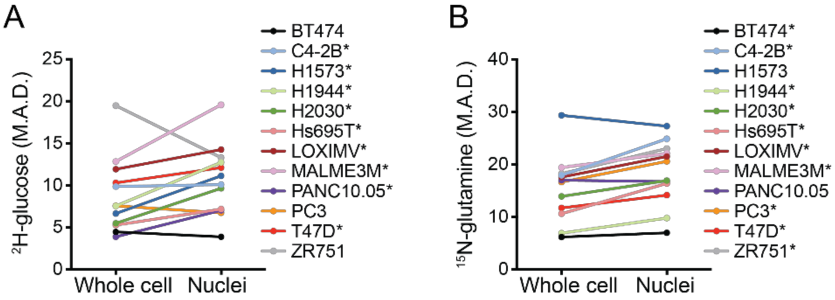

Figure S2. Heterogeneity of MIMS metabolic labeling increases with improved intracellular resolution in cultured cancer cells, related to Figure 1. Cultured cancer cell lines were labeled with BrdU, ^{2}H-glucose, and ^{15}N-glutamine for 12 h prior to MIMS analysis. Regions of interest (ROI) were selected for the BrdU-labeled cells. For each cell, the entire circumference was used to generate an ROI and the ^{81}Br image was used to select ROI corresponding to the BrdU-labeled nucleus. The median absolute deviation (M.A.D.) metric of dispersion was calculated for the population of BrdU-labeled cells and the corresponding population of BrdU-labeled nuclei. **(A)** M.A.D. for ^{2}H-glucose labeling. * denotes those cell lines (9/12) in which the M.A.D. increased in the nuclei relative to corresponding whole cells. **B)** M.A.D. for ^{15}N-glutamine labeling. * denotes those cell lines (10/12) in which the M.A.D. increased in the nuclei relative to corresponding whole cells.

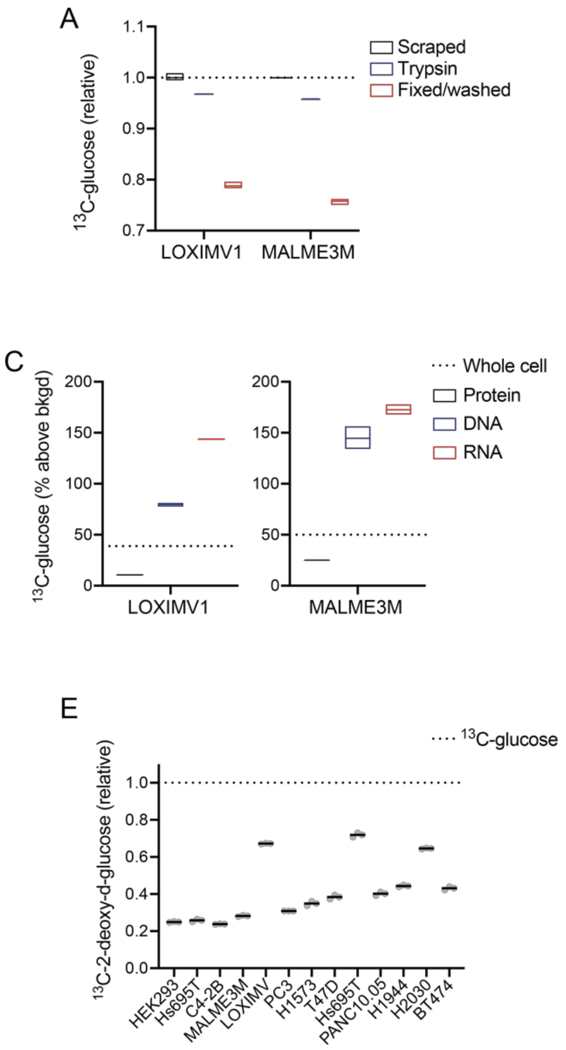

Figure S3. Incorporation of glutamine and glucose label into multiple anabolic pathways of fixable biomass by cancer cell lines, related to Figure 1. For each experiment, ^{13}C-glucose, ^{15}N-glutamine, and/or ^{13}C-2-deoxy-d-glucose were administered to cultured cancer cell lines for 12 hours. Label incorporation was measured with isotope ratio mass spectrometry (IRMS). **(A)** The effect of sequential trypsinization (blue) then fixation and alcohol washing (red) on ^{13}C-glucose labeling was quantified and compared to scraped LOXIMV1 and MALME3M cells as a reference. Note y-axis intersect starts at 0.7 (70% of scraped cell mean). **(B)** The effect of sequential trypsinization (blue) then fixation and alcohol washing (red) on ^{15}N-glutamine labeling was quantified and compared to scraped LOXIMV1 and MALME3M cells as a reference. Note y-axis intersect starts at 0.7 (70% of scraped cell mean). **(C)** Protein (gray), DNA (blue), and RNA (red) was extracted from ^{13}C-glucose labeled cells and the signal in the respective fractions compared to whole cells (dashed line). **(D)** Protein (gray), DNA (blue), and RNA (red) was extracted from ^{15}N-glutamine labeled cells and the signal in the respective fractions compared to whole cells (dashed line). **(E)** For each cell line, the ^{13}C-2-deoxy-d-glucose signal was quantified relative to the ^{13}C-glucose signal (dashed line) after cultured cells were labeled in parallel. The ^{13}C-2-deoxy-d-glucose signal, which represents glucose uptake only, was consistently lower than ^{13}C-glucose, which is both taken up and metabolized via catabolic and anabolic pathways. Box plots (A-D) show mean line, max/min, 3 technical replicates. Dot plot in E shows mean line, 3 technical replicates.

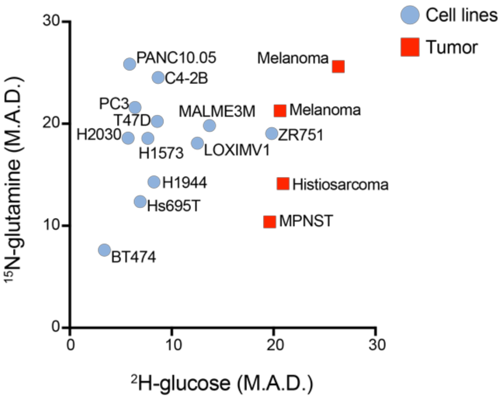

Figure S4. Heterogeneity of murine tumors relative to cultured cancer cell lines, related to Figures 1-2. The median absolute deviation (M.A.D.) is a robust, non-parametric representation of dispersion that is resilient to outliers yet inclusive of all data points. We utilized the M.A.D. metric of dispersion to describe and compare MIMS labeling distributions within and between cell types and tumors. This graph compares the M.A.D. for the analyzed cell lines (Figure 1, blue circles) and our initial round of tumors (Figure 2, red squares). The tumors generally displayed greater heterogeneity than the cultured cells lines, particularly with respect to ^{2}H-glucose utilization.

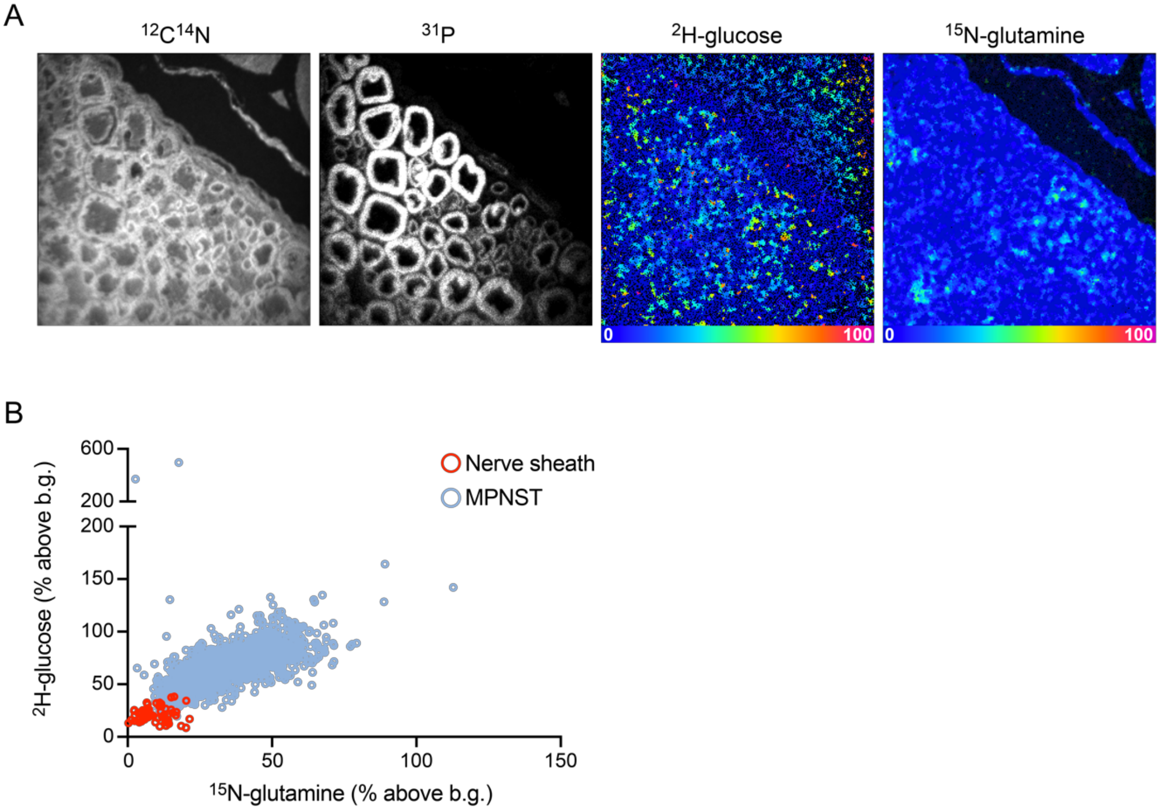

Figure S5. Heterogeneous substrate labeling in MPNST relative to normal peripheral nerve sheath cells, related to Figures 3. (A) Peripheral nerve shown in cross section. The myelin sheaths containing cells of origin for MPNST are evident in the ^{31}P image. HSI images demonstrate scattered puncta of low-level labeling. **(B)** Dot plot of the quantitative data for A (red circles) and shown relative to a representative MPNST. The MPNST demonstrates both increased absolute labeling and increased heterogeneity of labeling.

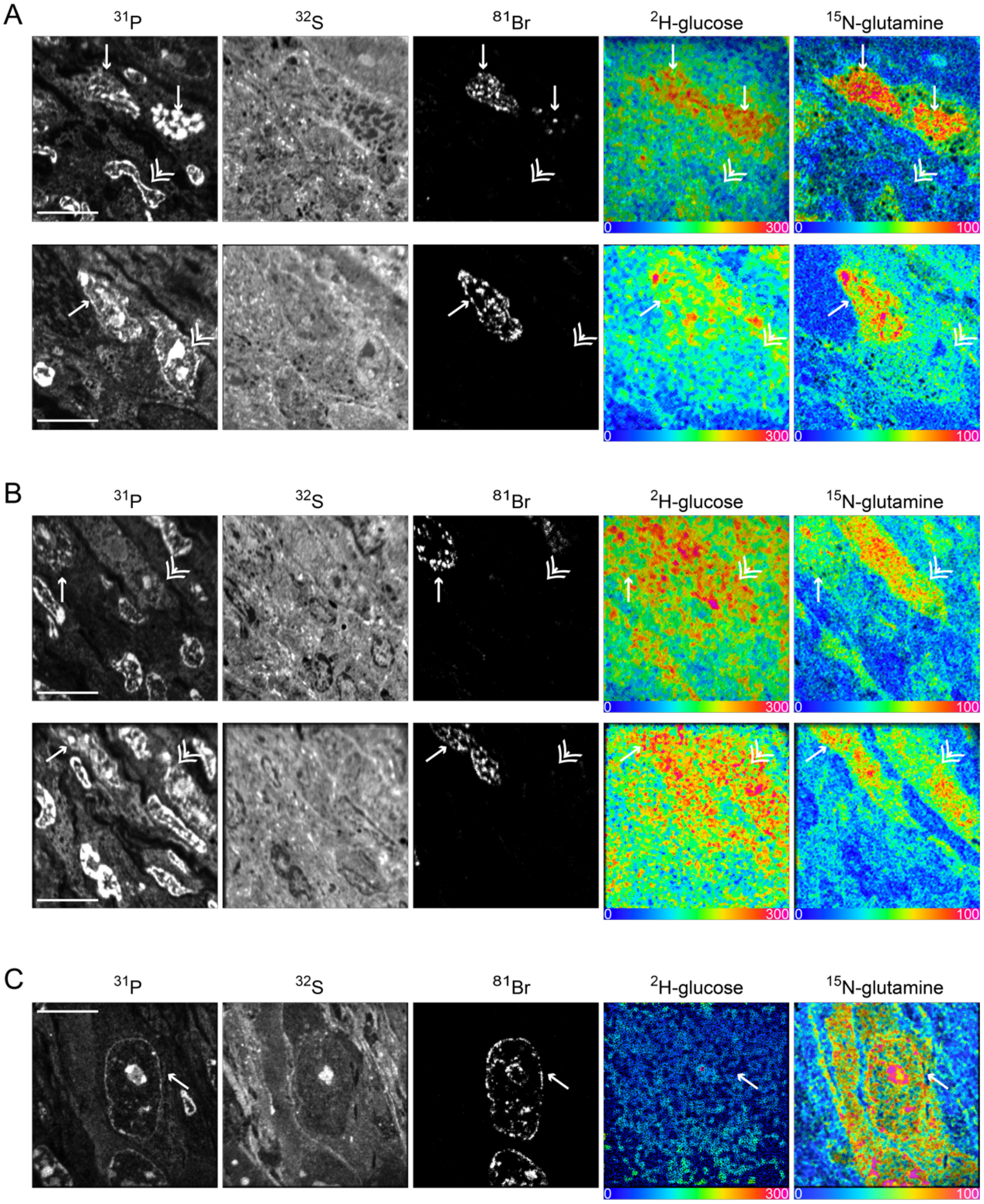

Figure S6. Metabolic heterogeneity of murine malignant peripheral nerve sheath tumors in *Nf1$^{+/-}$/Tp53$^{+/-}$/Suz12$^{+/-}$* mice, related to Figure 3. (A) Representative examples of highly labeled, BrdU$^+$ cells (small arrows) near less intensely labeled BrdU$^-$ cells (hatched arrow heads), examples where the putative divided cells demonstrated augmentation of labeling. **(B)** Representative examples of highly labeled, but BrdU$^-$ cells (hatched arrow head), comparable to BrdU$^+$ cells (small arrows). **(C)** Representative example of discordant ^{2}H-glucose (low) and ^{15}N-glutamine (high) labeling in BrdU$^+$ cells (small arrow).

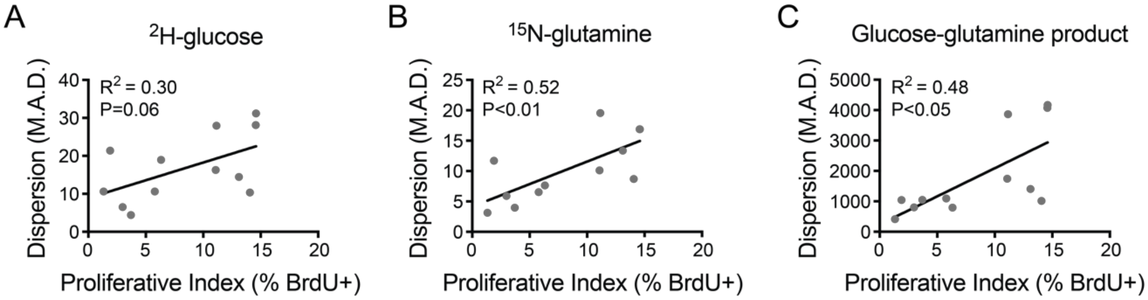

Figure S7. Metabolic heterogeneity of murine malignant peripheral nerve sheath tumors in *Nf1*$^{+/-}$ */Tp53*$^{+/-}$*/Suz12*$^{+/-}$ mice as a function of proliferative index, related to Figure 3. Correlations between the degree of heterogeneity (M.A.D.) in the BrdU+ tumor cell populations and the overall proliferative index for the respective tumor. **(A)** ^{2}H-glucose dispersion versus proliferative index. **(B)** ^{15}N-glutamine dispersion versus proliferative index. **(C)** Dispersion of the glucose-glutamine product versus proliferative index. Similar findings as shown here in A-C were noted when the analysis included all cells (BrdU+ and BrdU-) as shown in Figure 3.

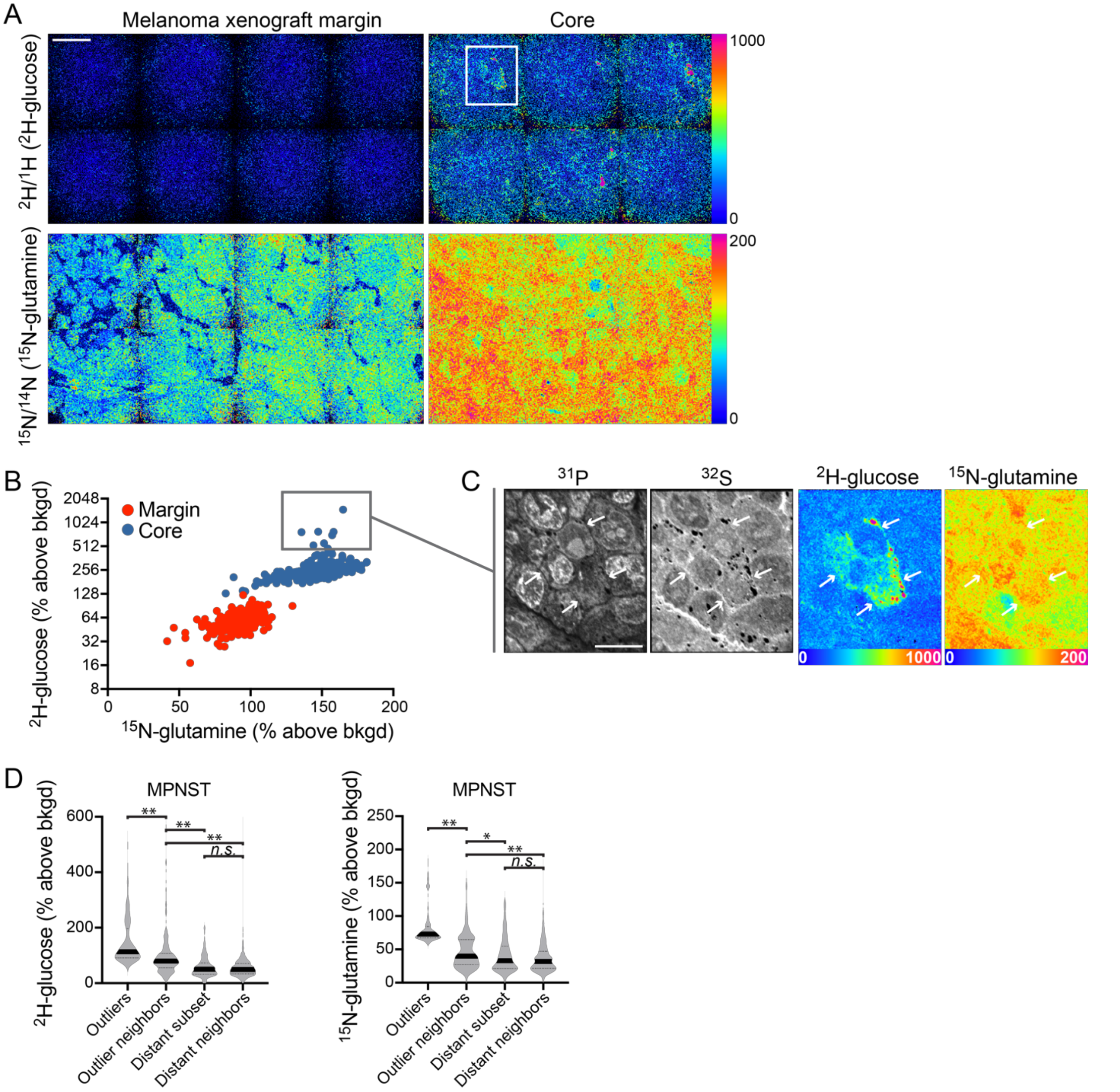

Figure S8. Metabolic clustering in melanoma xenograft and MPNST, related to Figure 5. (A) Xenograft model administered ^{2}H-glucose and ^{15}N-glutamine for 5 days, twice daily I.P. dosing. Left: region from tumor margin. Right: region from tumor core. Scale=20μm. **(B)** Dot plot of ^{2}H-glucose and ^{15}N-glutamine labeling, color-coded according to margin (red) versus core (blue). Note: Y-axis is $\log_2$ scale. **(C)** In the core, there were scattered clusters of glucose avid cells. Higher resolution images of the region highlighted by the white box in (A) are shown. Scale=20μm. **(D)** MPNST tumors (pooled analysis of 5 untreated tumors) analyzed for evidence of more subtle metabolic clustering. Labeling outliers (n=159 ^{15}N-glutamine Tukey outliers; n=145 ^{2}H-glucose Tukey outliers) were identified and compared to adjacent cells ("Outlier neighbors"). Subsets of tumor cells distant to the outliers were selected as controls (generated randomly by JMP 14.0) and compared to their adjacent cells. The "Distant" subset (n=159) demonstrated similar labeling as the population of adjacent cells ("Distant neighbors"). In contrast, cells adjacent to the highly labeled outliers were less labeled than the outlier population, but slightly higher than the more distant populations. This suggests that the outlier cells were anchoring clusters of cells that tended to be more highly labeled, either due to genetic effects or to non-cell autonomous effects of a shared microenvironment. Violin plots with median line: *$p<0.05$; **$p<0.0001$; *n.s.*=not significant. A Kruskal Wallis Test (nonparametric) was applied followed by Dunn's correction for the multiple comparisons indicated in the graph.

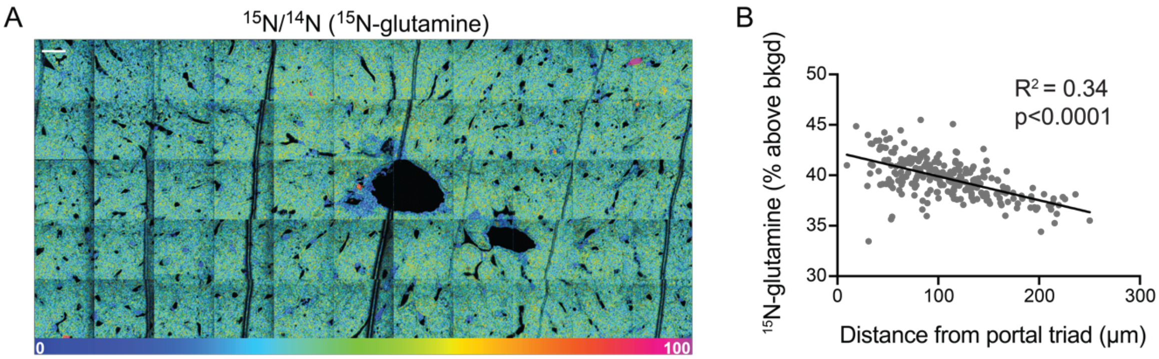

Figure S9. Liver zonation effect on ^{15}N-glutamine labeling (related to Figure 5). (A) Representative ^{15}N-glutamine MIMS image, demonstrating increased ^{15}N-labeling near the portal zone of the liver. Scale=20µm. **(B)** Representative dot plot of ^{15}N-glutamine labeling of hepatocytes as a function of distance from portal triad.

Supplemental Table:

Table S1. Correlation between glucose and glutamine utilization by malignant peripheral nerve sheath tumors (MPNST, related to Figure 3). MIMS revealed a statistically consistent, but variable degree, of correlation between glucose (^2H-labeling) and glutamine (^{15}N-labeling) utilization.

Tumor	Spearman Rho	p-value
Untreated tumors		
1	0.6980	<0.0001
2	0.6497	<0.0001
3	0.5582	<0.0001
4	0.8042	<0.0001
5	0.5492	<0.0001
JQ1/PD901		
1	0.7432	<0.0001
2	0.8646	<0.0001
3	0.5410	<0.0001
4	0.2562	<0.0001
5	0.6757	<0.0001
6	0.4857	<0.0001
7	0.5929	<0.0001

Transparent Methods:

Cell Lines

Human cancer cell lines utilized in this study, included: breast cancer lines BT474 (purchased from ATCC), T47D (ATCC), ZR751 (ATCC); prostate cancer lines C42B (ATCC), PC3 (ATCC); lung cancer lines H1573 (ATCC), H1944 (ATCC), H2030 (ATCC); melanoma lines Hs695T (ATCC), LOXIMVI (NCI/DCTD), MALME3M (ATCC); and pancreatic cancer line PANC10.05 (ATCC).

Mice

Studies were approved by the Brigham and Women's Hospital Institutional Animal Care and Use Committee. We utilized two genetically engineered mouse models of malignant peripheral nerve sheath tumors (MPNST), both of which have previously been described(Cichowski et al., 1999; De Raedt et al., 2014): the *Nf1*$^{+/-}$*/P53*$^{+/-}$ and the *Nf1*$^{+/-}$*/P53*$^{+/-}$*/Suz12*$^{+/-}$ mouse models, inclusive of males and females. MPNSTs were identified in adult mice within the first year of life by their appearance as palpable tumors in the subcutaneous tissues and then verified histologically by their stereotypical appearance consisting of fascicles of spindle cells with elongated and wavy nuclei. For the treatment study of tumors arising in the *Nf1*$^{+/-}$*/P53*$^{+/-}$*/Suz12*$^{+/-}$ model, we utilized a previously validated and molecularly targeted therapeutic combination of JQ1 and PD-0325901(De Raedt et al., 2014). JQ1 (45 mg/kg) was administered by daily intraperitoneal injection in a 10% (2-hydroxypropyl)-β-cyclodextrin solution (C0926, Sigma). PD-0325901 was administered by daily oral gavage at 1.5 mg/kg (vehicle (0.5% (w/v) methylcellulose solution with 0.2% (v/v) polysorbate 80 (Tween 80)).

For melanoma xenografting, nude mice were inoculated subcutaneously with 3×10^6 human melanoma cells (A375) as previously described(Maertens et al., 2013). Tumor volumes were calculated by measuring length and width of the lesions and with the formula $[(\text{length}) \times (\text{width})^2 \times 0.52]$. When tumors were growing in log phase, mice were labeled with the stable isotope tracer cocktail.

Mosaic Yap activation in the liver was achieved using a previously described mouse model(Yimlamai et al., 2014). A hepatocyte-specific Cre-expressing adeno-associated virus (AAV-Cre) was intravenously delivered to mice containing inducible alleles for tetracycline-inducible expression of YAP S127A (a constitutive-active form of YAP) and a reverse tetracycline-controlled transactivator (TetOYap). Subsequent doxycycline administration leads to mosaic activation of transgenic YAP. Genetically recombined hepatocytes express tdTomato, which was detected with an anti-RFP antibody (Rockland Antibodies).

For acquisition of tissues, mice were administered a lethal dose of ketamine/xylazine, the chest was opened and the mice were perfused with PBS followed by 4% paraformaldehyde via cannulation of the left ventricle. Tissues were excised and placed in 4% paraformaldehyde at 4°C for additional fixation overnight. The tumors were excised including the surrounding tissue and cut into smaller slices extending from the tumor margin into the tumor core to facilitate penetration of fixative. Regions from the tumor margin and tumor core were used for embedding and subsequently processing for MIMS analysis. Regions of grossly evident necrosis were avoided.

Multi-isotope imaging mass spectrometry (MIMS)

Stable isotope tracers were purchased from Cambridge Isotope Laboratories. Bromodeoxyuridine (BrdU) was purchased from Sigma. For cell culture experiments, the culture medium (RPMI-1640, Corning) was supplemented with BrdU (10 μM), ^{15}N-glutamine (30 mg/L), and ^{2}H-glucose (200 mg/L). For mouse studies, a labeling cocktail consisting of BrdU (500μg/dose), ^{2}H-glucose (50mg/dose), and ^{15}N-glutamine (5mg/dose) was administered by intraperitoneal injection every 12 hours. Fixed (4% paraformaldehyde) cell pellets or mouse tissues were embedded in EPON, sectioned to 0.5 microns, and mounted on silicon wafers. Samples were gold-coated and analyzed with the NanoSIMS 50L (CAMECA) at the Brigham and Women's Hospital Center for NanoImaging. ^{15}N-glutamine labeling was quantified by measuring the $^{12}C^{15}N^-/^{12}C^{14}N^-$ ratio as previously described(Kim et al., 2014; Steinhauser et al., 2012). ^{2}H-glucose labeling was quantified by measuring the $^{12}C_2{}^2H^-/^{12}C_2{}^2H^-$ as previously described(Guillermier et al., 2017a; Guillermier et al., 2017b). Sections of cell pellets and tumors were analyzed in automated chain analysis mode, with each tile acquired at 256 x 256 pixels, 50 μm x 50 μm field size. This automated approach augmented throughput by facilitating data collection during off hours and also allowed for visualization of larger swaths of a given section. Images were viewed and processed, using a custom plugin to ImageJ (OpenMIMS 3.0: https://github.com/BWHCNI/OpenMIMS). Regions of interest (ROI) were selected for nucleated tumor cells utilizing $^{12}C^{14}N^-$, $^{31}P^-$, and $^{32}S^-$ mass images. When automated analyses are viewed as a mosaic image of sequential tiles, some edge artifacts become evident at the margins between adjacent tiles. Cells that were

not well visualized due to insufficient ion counts or due to imprecise alignment of adjacent tiles were excluded from the analysis. Because the images are derived from measurements from the uppermost atomic layers of the section, however, the chain analyses leave abundant tissue in the z-axis to reanalyze specific areas of interest when additional imaging resolution is desired or when crucial features are captured at the juncture between two adjacent fields.

Isotope ratio mass spectrometry (IRMS)

For the analyses detailed in Figure S3, pooled cells or cellular fractions were analyzed by IRMS, as previously described(Hosios et al., 2016; Kim et al., 2014; Zhang et al., 2018). Briefly, samples were pipetted into tin cups (Elementar) and dried at 60C. Prior to analysis, each tin cup was crushed and sequentially introduced to an elemental analyzer (Vario PYRO Cube, Elementar) coupled to an IRMS (Isoprime 100, Elementar). Tuning was confirmed with urea standards, and samples obtained from unlabeled cells were used as an additional control for the natural background ratio. ^{15}N-labeling was detected as signal above the natural $^{15}N/^{14}N$ ratio ($^{15}N/^{14}N = 0.37\%$). ^{13}C-labeling was detected as signal above the natural $^{13}C/^{12}C$ ratio ($^{13}C/^{12}C = 1.11\%$).

Statistics

Descriptive statistics, such as the median absolute deviation (M.A.D. = median (|x-median(x)|).), were generated by JMP Pro 13.0 or 14.0 (SAS). Data were graphed and statistical analyses performed with JMP or Prism 7/8 (Graphpad). Normality was assessed using a Shapiro-Wilk test. For comparisons of two normally distributed data sets, we performed a two-sided t test. For non-parametric data consisting of two groups, we performed a Mann-Whitney test.

Supplemental References:

Cichowski, K., Shih, T.S., Schmitt, E., Santiago, S., Reilly, K., McLaughlin, M.E., Bronson, R.T., and Jacks, T. (1999). Mouse models of tumor development in neurofibromatosis type 1. Science *286*, 2172-2176.

De Raedt, T., Beert, E., Pasmant, E., Luscan, A., Brems, H., Ortonne, N., Helin, K., Hornick, J.L., Mautner, V., Kehrer-Sawatzki, H., *et al.* (2014). PRC2 loss amplifies Ras-driven transcription and confers sensitivity to BRD4-based therapies. Nature *514*, 247-251.

Guillermier, C., Fazeli, P.K., Kim, S., Lun, M., Zuflacht, J.P., Milian, J., Lee, H., Francois-Saint-Cyr, H., Horreard, F., Larson, D., *et al.* (2017a). Imaging mass spectrometry demonstrates age-related decline in human adipose plasticity. JCI Insight *2*, e90349.

Guillermier, C., Poczatek, J.C., Taylor, W.R., and Steinhauser, M.L. (2017b). Quantitative imaging of deuterated metabolic tracers in biological tissues with nanoscale secondary ion mass spectrometry. International journal of mass spectrometry *422*, 42-50.

Hosios, A.M., Hecht, V.C., Danai, L.V., Johnson, M.O., Rathmell, J.C., Steinhauser, M.L., Manalis, S.R., and Vander Heiden, M.G. (2016). Amino Acids Rather than Glucose Account for the Majority of Cell Mass in Proliferating Mammalian Cells. Dev Cell *36*, 540-549.

Kim, S.M., Lun, M., Wang, M., Senyo, S.E., Guillermier, C., Patwari, P., and Steinhauser, M.L. (2014). Loss of white adipose hyperplastic potential is associated with enhanced susceptibility to insulin resistance. Cell Metab *20*, 1049-1058.

Maertens, O., Johnson, B., Hollstein, P., Frederick, D.T., Cooper, Z.A., Messiaen, L., Bronson, R.T., McMahon, M., Granter, S., Flaherty, K., *et al.* (2013). Elucidating distinct roles for NF1 in melanomagenesis. Cancer discovery *3*, 338-349.

Steinhauser, M.L., Bailey, A.P., Senyo, S.E., Guillermier, C., Perlstein, T.S., Gould, A.P., Lee, R.T., and Lechene, C.P. (2012). Multi-isotope imaging mass spectrometry quantifies stem cell division and metabolism. Nature *481*, 516-519.

Yimlamai, D., Christodoulou, C., Galli, G.G., Yanger, K., Pepe-Mooney, B., Gurung, B., Shrestha, K., Cahan, P., Stanger, B.Z., and Camargo, F.D. (2014). Hippo pathway activity influences liver cell fate. Cell *157*, 1324-1338.

Zhang, Y., Federation, A.J., Kim, S., O'Keefe, J.P., Lun, M., Xiang, D., Brown, J.D., and Steinhauser, M.L. (2018). Targeting nuclear receptor NR4A1-dependent adipocyte progenitor quiescence promotes metabolic adaptation to obesity. J Clin Invest *128*, 4898-4911.
